# The real-world safety profile of ranolazine: pharmacovigilance analysis of the FAERS database

**DOI:** 10.3389/fphar.2025.1702875

**Published:** 2025-11-10

**Authors:** Zujun Wen, Xiang Liu, Yanbin Yi, Gengliang Lin, Peng Liu, Tingting Zhang

**Affiliations:** 1 Department of pharmacy, Heyuan People’s Hospital, Guangdong Provincial People’s Hospital Heyuan Hospital, Heyuan, China; 2 Department of Pharmacy, The First Affiliated Hospital of Chongqing Medical and Pharmaceutical College, Chongqing, China; 3 Department of Spine Surgery, Heyuan People’s Hospital, Guangdong Provincial People’s Hospital Heyuan Hospital, Heyuan, China; 4 Department of Pharmacy, Dazhou Central Hospital, Dazhou, Sichuan, China

**Keywords:** adverse events, ranolazine, FDA adverse event reporting system (FAERS), disproportionality analysis, real-world data analysis

## Abstract

**Background:**

Ranolazine, a piperazine derivative, is used as a second-line treatment for individuals with stable or poorly managed chronic angina as well as for those whose chronic angina is not improving with other medications. However, there is still a lack of real-world evidence on the long-term safety of its adverse events (AEs), which calls for constant supplementation.

**Methods:**

This study conducted disproportionality analysis to evaluate the safety of ranolazine in clinical practice by utilizing data from the FDA Adverse Event Reporting System (FAERS). Reporting Odds Ratio, Proportional Reporting Ratio, Multi-item Gamma Poisson Shrinker and Bayesian Confidence Propagation Neural Network were used to identify signals of possible adverse events linked to ranolazine. Additionally, the Weibull distribution was implemented to simulate how the frequency of adverse events changed over time.

**Results:**

Positive signals were found for adverse events listed on the medication label, including dizziness, nausea, asthenia, constipation, headache, palpitations, tinnitus, abdominal pain, dry mouth, vomiting, peripheral edema, dyspnea and hypotension. Additionally, potential side effects not included on the label were also identified, including chest pain, cardiac disorder, electrocardiogram qt prolonged, hypertension, seizure, tremor, atrial fibrillation, coronary artery disease, pulmonary embolism, myoclonus and myocardial infarction. The significance of tracking adverse events was underlined, especially during the first month after the start of treatment.

**Conclusion:**

Our study has confirmed certain known adverse effects and identified other potential hazards by providing preliminary safety data on the practical use of ranolazine. The results present vital safety information for physicians prescribing ranolazine. Notably, this study only identifies safety signals and does not establish causal relationships between ranolazine and the observed adverse events.

## 1 Introduction

Ranolazine, a piperazine derivative, is used as second-line therapy in patients with stable or poorly controlled chronic angina and in patients with chronic angina unresponsive to other drugs (Nash and Nash, 2008). Stable angina pectoris (AP) is characterized by recurrent episodes of myocardial ischemia, manifesting when the heart’s oxygen requirements outstrip its available supply (Abrams, 2005; Hasenfuss and Maier, 2008). Angina, a common symptom in patients with chronic coronary syndromes (CCS), imposes significant limitations on patients’ functional capacity and adversely impacts their quality of life (QoL) (Brorsson et al., 2002; Tousoulis et al., 2013). The central objective in chronic coronary syndrome (CCS) management is to alleviate angina pectoris (AP) and exercise-induced ischemia, prevent cardiovascular events, and enhance symptom control, prognosis, and quality of life (QoL) through guideline-directed pharmacotherapy, revascularization, and lifestyle interventions (Knuuti et al., 2019; Cattaneo et al., 2015).

Ranolazine improves diastolic tension, myocyte relaxation, and angina symptoms by blocking late sodium channels and avoiding intracellular calcium excess (Wilson et al., 2009). Without appreciably altering heart rate or blood pressure, its special inhibitory activity in the late phase of the inward sodium current in cardiac myocytes lowers myocardial oxygen consumption (Bertero et al., 2021; Chaitman et al., 2004). According to the treatment algorithm proposed by Manolis et al. (2021), ranolazine is a suitable treatment option for all hemodynamic groups and all clinical scenarios that are presented, including diabetes mellitus, microvascular angina, atrial fibrillation, heart failure with reduced ejection fraction, significant conduction abnormalities, and chronic obstructive pulmonary disease. The general population safety profile of ranolazine could not be well reflected due to the strict inclusion and exclusion criteria of clinical studies. Besides that, there are currently insufficient real-world safety data for ranolazine. Therefore, it is critically necessary to conduct a comprehensive evaluation of ranolazine’s safety while including a wide range of real-world data.

The FDA developed the publicly accessible FAERS database, which collects reports of adverse drug events from all over the world. It includes a wealth of real-world data and a wide geographic coverage of AE records from consumers, registered nurses, practicing physicians, pharmacists, and other healthcare professionals, among others (Vogel et al., 2020; Feng et al., 2022). In this work, we used the FAERS database and a number of disproportionality analysis approaches to evaluate the possible side effects linked to ranolazine. Furthermore, we investigated variations by age and gender and carried out an extensive assessment of the time to onset of adverse effects. The findings could provide clinicians with guidance on the safe administration of this medication.

## 2 Materials and methods

### 2.1 Data sources, management, and study design

**FIGURE 1:**
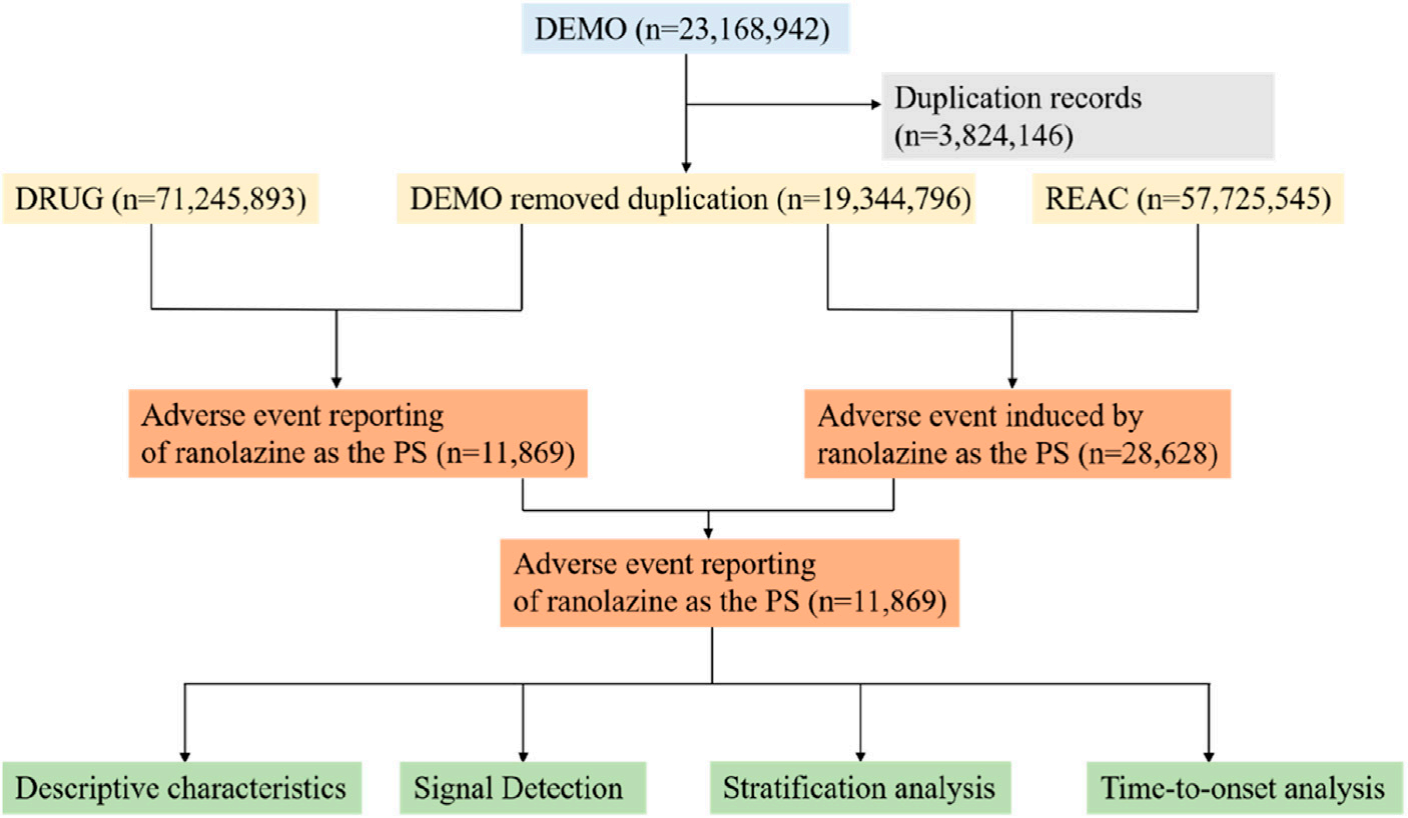
Data collection flow chart for adverse events of ranolazine. DEMO, demographic and administrative information; DRUG, drug information; REAC, coded for the adverse events; PS, primary suspect.

Our research conducted a post-marketing pharmacovigilance analysis of ranolazine using the FAERS database, which collects spontaneous adverse event reports from patients, pharmaceutical companies, and medical professionals across different geographical areas. The search period ran from Q1 2006 to Q2 2025, and all adverse event reports where ranolazine was identified as the major suspected medication were included in the study. The flow chart of this study, which shows the steps involved in data extraction, processing, and analysis, is presented in Figure 1.

Subsequently, we standardized the adverse events and deduplicated them in accordance with FDA guidelines. Reports with the same case identification (CASEID) were kept along with the report with the most recent FDA reception date (FDA_DT); reports with the highest PRIMARYID (the unique identifier assigned to each report) were kept together when the values of CASEID and FDA_DT matched. After then, the Medical Dictionary for Regulatory Activities (MedDRA 26.1), which categorizes events at the system organ class (SOC) and preferred term (PT) levels, was used to standardize the nomenclature for adverse events.

### 2.2 Statistical analysis

The characteristics of adverse event reports linked to ranolazine were described using descriptive analysis. Reporting Odds Ratio (ROR) (Rothman et al., 2004), Proportional Reporting Ratio (PRR) (Evans et al., 2001), Multi-item Gamma Poisson Shrinker (MGPS) (Ri et al., 2010), and Bayesian Confidence Propagation Neural Network (BCPNN) (Ang et al., 2016) were used to identify signals of possible adverse reactions linked to ranolazine. The PRR, a tool for assessing relative risk, is recognized for its great sensitivity, but it’s also susceptible to false-positive results, especially in situations with a limited number of cases (Jiang et al., 2024). The ROR serves as a more reliable estimate technique of rate or hazard ratio that can adjust for bias in situations when there are few adverse event reports (Sakaeda et al., 2013). Both BCPNN and MGPS leverage Bayesian statistical principles. BCPNN is valued for its ability to deliver relatively stable results even with a small number of reports (Shu et al., 2025). Whereas MGPS is particularly adept at identifying signals of unexpected or rare events (Sakaeda et al., 2014). To ensure robust and reliable findings, we only retained adverse event signals that were statistically significant across all four aforementioned methods (ROR, PRR, BCPNN, and MGPS) for subsequent analysis. This integrative strategy leverages the complementary strengths of each algorithm, enhancing detection efficiency while mitigating false positives through cross-validation. It also facilitates the identification of rare adverse reactions. Supplementary Table S1 contains comprehensive two-by-two contingency tables. And these disproportionality assessments’ formulae and criteria are described in Supplementary Table S2. The initial onset time of ranolazine-related adverse events was determined by the time gap between the incidence of adverse events (as documented in the DEMO file) and the start of ranolazine therapy (as documented in the THER file). The Weibull distribution was implemented to simulate how the frequency of adverse events changed over time. All analyses were performed using R software version 4.2.2.

## 3 Results

### 3.1 Baseline characteristics of study participants

Over the course of the research period (Q1 2006–Q2 2025), 23,168,942 reported cases were retrieved from the FAERS database. Following deduplication, this analysis ultimately included 28,628 adverse events linked to ranolazine and 11,869 reports of adverse events related to ranolazine (Figure 1). In Table 1, the clinical characteristics of adverse event reports with ranolazine are presented. Among these reports were 6,159 (51.9%) from men, 5,053 (42.6%) from women, and 657 (5.5%) whose gender information was missing. Of all the reports, 42.4% were from people aged over 65 years old. The United States, England, Germany, Italy and Canada were the top five countries with the highest number of reports. According to reporting years, 2015 had the highest percentage of reports (20.7%), followed by 2018 (13.7%), 2017 (12.1%), 2016 (11.2%), and 2019 (10.1%). Comprehensive information on ranolazine -related adverse event reports can be found in Table 1.

**TABLE 1:** Clinical characteristics of ranolazine adverse event reports from the FAERS database (Q1 2006 – Q2 2025).

Characteristics	Case numbers	Case proportion (%)
Number of events	11,869	
Gender		
Male	6,159	51.9%
Female	5,053	42.6%
Miss	657	5.5%
Age(years)		
<18	169	1.4%
18–65	2,936	24.7%
65–85	5,034	42.4%
>85	816	6.9%
Miss	2,914	24.6%
Top 5 reported countries		
United States	9,850	83.0%
England	565	4.8%
Germany	315	2.7%
Italy	220	1.9%
Canada	130	1.1%
Reporter		
Healthcare professional	4,436	37.4%
Non-healthcare professional	7,221	60.8%
Miss	212	1.8%
Reporting year		
2006	69	0.6%
2007	193	1.6%
2008	86	0.7%
2009	114	1.0%
2010	129	1.1%
2011	99	0.8%
2012	127	1.1%
2013	329	2.8%
2014	918	7.7%
2015	2,457	20.7%
2016	1,327	11.2%
2017	1,434	12.1%
2018	1,622	13.7%
2019	1,199	10.1%
2020	368	3.1%
2021	245	2.1%
2022	213	1.8%
2023	310	2.6%
2024	398	3.4%
2025	232	2.0%

### 3.2 Distribution of adverse events at the system organ class (SOC) level

**FIGURE 2:**
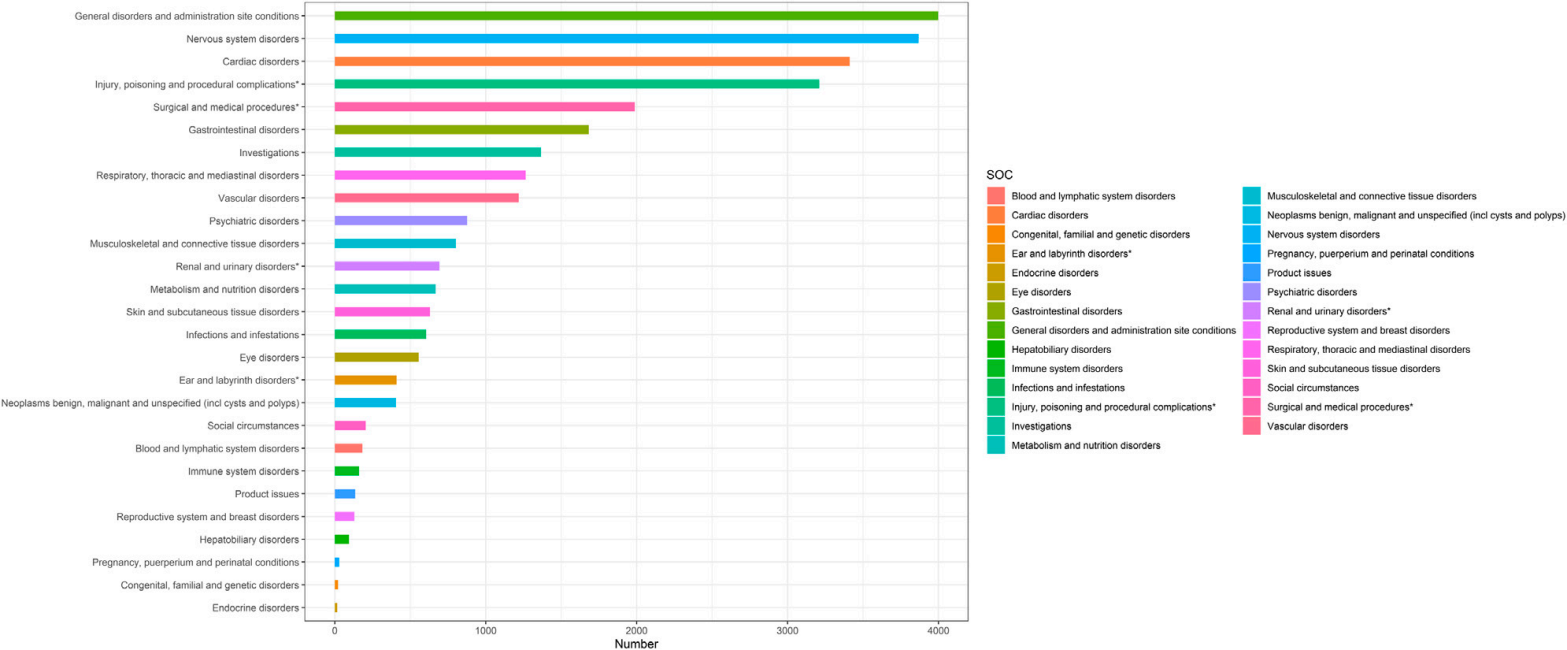
System organ classes (SOC) distribution.

Based on statistical analysis, we discovered that 27 organ systems were the focus of ranolazine-induced adverse events. As illustrated in Table 2, significant results were identified in a number of categories, including nervous system disorders, cardiac disorders, injury, poisoning and procedural complications, surgical and medical procedures, vascular disorders, renal and urinary disorders, ear and labyrinth disorders, and social circumstances. Figure 2 shows the distribution of adverse events at the SOC level.

**TABLE 2:** Signal strength of ranolazine AEs across system organ classes (SOC) in the FAERS database.

System organ class (SOC)	Case numbers	ROR (95%CI)	PRR (χ^2^)	EBGM (EBGM05)	IC (IC025)
Eye disorders	556	0.97 (0.9–1.06)	0.97 (0.39)	0.97 (0.9)	−0.04 (−0.16)
Investigations	1,366	0.77 (0.73–0.81)	0.78 (90.37)	0.78 (0.74)	−0.36 (−0.44)
Product issues	135	0.28 (0.24–0.33)	0.29 (245.5)	0.29 (0.24)	−1.81 (−2.05)
Endocrine disorders	16	0.22 (0.13–0.35)	0.22 (45.15)	0.22 (0.13)	−2.2 (−2.83)
Vascular disorders*	1,219	2.05 (1.94–2.17)	2.01 (628.54)	2.01 (1.89)	1 (0.92)
Social circumstances*	205	1.53 (1.33–1.75)	1.52 (36.93)	1.52 (1.33)	0.61 (0.4)
Hepatobiliary disorders	94	0.35 (0.29–0.43)	0.36 (110.82)	0.36 (0.29)	−1.49 (−1.78)
Nervous system disorders*	3,868	1.7 (1.64–1.76)	1.6 (961.44)	1.6 (1.55)	0.68 (0.63)
Immune system disorders	161	0.5 (0.43–0.59)	0.51 (78.51)	0.51 (0.43)	−0.98 (−1.21)
Cardiac disorders*	3,412	5.08 (4.9–5.26)	4.59 (9,816.82)	4.58 (4.42)	2.2 (2.14)
Psychiatric disorders	877	0.53 (0.5–0.57)	0.55 (343.49)	0.55 (0.51)	−0.86 (−0.96)
Musculoskeletal and connective tissue disorders	802	0.53 (0.49–0.56)	0.54 (331.62)	0.54 (0.5)	−0.89 (−0.99)
Congenital, familial and genetic disorders	21	0.24 (0.16–0.38)	0.25 (48.85)	0.25 (0.16)	−2.03 (−2.59)
Neoplasms benign, malignant and unspecified (incl cysts and polyps)	405	0.54 (0.49–0.59)	0.55 (157.4)	0.55 (0.49)	−0.87 (−1.02)
Metabolism and nutrition disorders	668	1.08 (1–1.17)	1.08 (4.04)	1.08 (1)	0.11 (0)
Reproductive system and breast disorders	130	0.51 (0.43–0.61)	0.52 (59.23)	0.52 (0.44)	−0.95 (−1.2)
Surgical and medical procedures*	1,988	5.41 (5.17–5.66)	5.1 (6,628.74)	5.09 (4.86)	2.35 (2.28)
General disorders and administration site conditions	3,998	0.77 (0.74–0.79)	0.8 (239.96)	0.8 (0.77)	−0.32 (−0.37)
Injury, poisoning and procedural complications*	3,210	1.08 (1.04–1.12)	1.07 (15.24)	1.07 (1.03)	0.09 (0.04)
Blood and lymphatic system disorders	183	0.37 (0.32–0.43)	0.38 (190.36)	0.38 (0.33)	−1.4 (−1.61)
Pregnancy, puerperium and perinatal conditions	28	0.23 (0.16–0.33)	0.23 (72.5)	0.23 (0.16)	−2.12 (−2.62)
Infections and infestations	605	0.39 (0.36–0.42)	0.4 (572.42)	0.4 (0.37)	−1.32 (−1.44)
Gastrointestinal disorders	1,683	0.67 (0.64–0.71)	0.69 (251.06)	0.69 (0.66)	−0.53 (−0.6)
Ear and labyrinth disorders*	409	3.34 (3.03–3.68)	3.31 (660.07)	3.3 (3)	1.72 (1.57)
Renal and urinary disorders*	693	1.29 (1.2–1.39)	1.28 (44.04)	1.28 (1.19)	0.36 (0.25)
Skin and subcutaneous tissue disorders	631	0.39 (0.36–0.43)	0.41 (578.48)	0.41 (0.38)	−1.3 (−1.41)
Respiratory, thoracic and mediastinal disorders	1,265	0.94 (0.88–0.99)	0.94 (5.35)	0.94 (0.89)	−0.09 (−0.17)

Abbreviation: Asterisks (*) indicate statistically significant signals in algorithm; ROR, reporting odds ratio; PRR, proportional reporting ratio; EBGM, empirical Bayesian geometric mean; EBGM05, the lower limit of the 95% CI, of EBGM; IC, information component; IC025, the lower limit of the 95% CI, of the IC; CI, confidence interval; AEs, adverse events.

### 3.3 Distribution of adverse events at the preferred term (PT) level

Frequencies of adverse events linked to ranolazine were graded, and positive signals were assessed. PTs with over 50 occurrences were our focus since they are often an excellent predictor of a drug-related adverse event. The adverse events listed on the medication’s label, which included dizziness, nausea, asthenia, constipation, headache, palpitations, tinnitus, abdominal pain, dry mouth, vomiting, peripheral edema, dyspnea and hypotension were proven to occur by the disproportionality analyses. Additionally, potential side effects that were not included on the label were also identified, including chest pain, cardiac disorder, electrocardiogram qt prolonged, hypertension, seizure, tremor, atrial fibrillation, coronary artery disease, pulmonary embolism, myoclonus and myocardial infarction. More detailed information on these findings is included in Table 3.

**TABLE 3:** Signal strength of reports of ranolazine administration at the preferred term level in the FAERS database.

PT	Case numbers	ROR (95%CI)	PRR (χ^2^)	EBGM (EBGM05)	IC (IC025)
Myocardial infarction*	660	8.04 (7.44–8.69)	7.88 (3,961.37)	7.85 (7.27)	2.97 (2.84)
Chest pain*	639	7.53 (6.96–8.15)	7.39 (3,527.42)	7.36 (6.81)	2.88 (2.75)
Angina pectoris	589	44.92 (41.36–48.78)	44.01 (24,242.33)	43.1 (39.68)	5.43 (5.21)
Stent placement	587	174.01 (159.83–189.46)	170.47 (91,193.14)	157.25 (144.43)	7.3 (6.83)
Dizziness	583	2.57 (2.37–2.79)	2.54 (548.29)	2.54 (2.34)	1.34 (1.22)
Cerebrovascular accident*	377	4.78 (4.32–5.3)	4.73 (1,110.59)	4.72 (4.27)	2.24 (2.08)
Intentional product use issue	293	7.49 (6.68–8.41)	7.43 (1,625.58)	7.4 (6.6)	2.89 (2.69)
Constipation	268	2.78 (2.46–3.13)	2.76 (301.32)	2.76 (2.44)	1.46 (1.28)
Cardiac disorder*	260	5.97 (5.28–6.75)	5.93 (1,063.44)	5.91 (5.23)	2.56 (2.36)
Diabetes mellitus	254	7.15 (6.31–8.09)	7.09 (1,326.01)	7.07 (6.25)	2.82 (2.61)
Seizure*	229	2.88 (2.53–3.28)	2.86 (278.21)	2.86 (2.51)	1.52 (1.31)
Hypotension	221	2.4 (2.1–2.74)	2.39 (178.28)	2.38 (2.09)	1.25 (1.05)
Hypoacusis*	208	9.3 (8.11–10.66)	9.24 (1,521.84)	9.2 (8.02)	3.2 (2.95)
Product use issue	203	2.43 (2.11–2.79)	2.42 (169.07)	2.42 (2.1)	1.27 (1.06)
Cardiac failure congestive*	196	5.04 (4.38–5.8)	5.01 (629.03)	5 (4.35)	2.32 (2.09)
Cardiac operation	193	52.19 (45.22–60.24)	51.85 (9,384.47)	50.57 (43.82)	5.66 (5.12)
Cardiac arrest	150	3.89 (3.31–4.57)	3.87 (319.48)	3.87 (3.29)	1.95 (1.69)
Syncope	145	3.1 (2.63–3.65)	3.09 (204.52)	3.08 (2.62)	1.62 (1.36)
Unevaluable event	143	4.04 (3.42–4.76)	4.02 (324.18)	4.01 (3.41)	2.01 (1.73)
Cardiac failure*	131	3.53 (2.97–4.19)	3.52 (235.97)	3.51 (2.96)	1.81 (1.53)
Atrial fibrillation*	127	2.81 (2.36–3.35)	2.8 (147.5)	2.8 (2.35)	1.49 (1.21)
Coronary artery disease*	122	8.24 (6.9–9.85)	8.21 (770.23)	8.18 (6.85)	3.03 (2.69)
Pulmonary embolism*	116	2.6 (2.16–3.11)	2.59 (113.14)	2.59 (2.16)	1.37 (1.08)
Cardiac pacemaker insertion	115	42.14 (35.02–50.7)	41.97 (4,506.16)	41.14 (34.19)	5.36 (4.66)
Dementia	112	8.97 (7.45–10.81)	8.94 (787.01)	8.91 (7.4)	3.16 (2.78)
Myoclonus	106	19.3 (15.93–23.38)	19.23 (1815.17)	19.06 (15.74)	4.25 (3.75)
Acute myocardial infarction	104	7.42 (6.12–9)	7.4 (573.42)	7.37 (6.08)	2.88 (2.51)
Catheterisation cardiac	103	50.75 (41.72–61.73)	50.57 (4,882.52)	49.36 (40.58)	5.63 (4.79)
Coronary artery bypass	101	36.5 (29.97–44.45)	36.38 (3,413.41)	35.75 (29.35)	5.16 (4.45)
Neoplasm malignant	101	3.28 (2.7–3.99)	3.27 (159.49)	3.27 (2.69)	1.71 (1.39)
Generalised tonic-clonic seizure	98	8.22 (6.74–10.03)	8.2 (617.25)	8.17 (6.7)	3.03 (2.64)
Chromaturia	96	8.99 (7.35–10.99)	8.96 (676.15)	8.93 (7.3)	3.16 (2.75)
Vascular graft	96	69.11 (56.37–84.72)	68.88 (6,209.79)	66.63 (54.35)	6.06 (5.01)
Coronary arterial stent insertion	95	41.04 (33.49–50.3)	40.91 (3,625.57)	40.12 (32.73)	5.33 (4.53)
Thrombosis*	94	2.51 (2.05–3.07)	2.5 (84.71)	2.5 (2.04)	1.32 (1)
Electrocardiogram qt prolonged	93	5.56 (4.53–6.82)	5.54 (345.58)	5.53 (4.51)	2.47 (2.1)
Wrong product administered	91	12.53 (10.19–15.4)	12.49 (956.49)	12.42 (10.11)	3.63 (3.16)
Coronary artery occlusion	90	13.78 (11.2–16.96)	13.74 (1,056.04)	13.65 (11.09)	3.77 (3.28)
Incorrect route of product administration	83	3.33 (2.68–4.13)	3.32 (134.58)	3.32 (2.67)	1.73 (1.38)
Deafness*	82	6.87 (5.53–8.54)	6.85 (408.79)	6.83 (5.5)	2.77 (2.36)
Intentional dose omission	82	7.74 (6.23–9.62)	7.72 (478.24)	7.7 (6.19)	2.94 (2.51)
Ventricular tachycardia*	77	10.06 (8.04–12.59)	10.04 (623.65)	9.99 (7.99)	3.32 (2.84)
Chronic obstructive pulmonary disease	71	2.91 (2.3–3.67)	2.9 (88.58)	2.9 (2.3)	1.54 (1.16)
Surgery	70	2.79 (2.21–3.53)	2.79 (80.29)	2.79 (2.2)	1.48 (1.1)
Implantable defibrillator insertion	69	117.04 (91.79–149.23)	116.76 (7,485.62)	110.42 (86.6)	6.79 (5.07)
Anaphylactic reaction	67	2.72 (2.14–3.45)	2.71 (72.49)	2.71 (2.13)	1.44 (1.05)
Bradycardia	66	2.65 (2.08–3.38)	2.65 (67.63)	2.65 (2.08)	1.4 (1.02)
Arterial occlusive disease	63	17.16 (13.39–22)	17.13 (948.81)	16.99 (13.26)	4.09 (3.4)
Status epilepticus	62	12.04 (9.38–15.46)	12.02 (622.63)	11.95 (9.31)	3.58 (2.98)
Blindness	59	3.18 (2.46–4.11)	3.18 (87.83)	3.17 (2.46)	1.67 (1.24)
Disability	50	5.52 (4.18–7.29)	5.51 (184.34)	5.5 (4.17)	2.46 (1.93)

Abbreviation: Asterisks (*) indicate unlabeled AEs; ROR, reporting odds ratio; PRR, proportional reporting ratio; EBGM, empirical Bayesian geometric mean; EBGM05, the lower limit of the 95% CI, of EBGM; IC, information component; IC025, the lower limit of the 95% CI, of the IC; CI, confidence interval; PT, preferred term.

### 3.4 Subgroup analysis

Subgroup analysis of ranolazine-related adverse events showed that, of the 50 most frequent adverse events that satisfied the positive signal criteria, severe neurological and cardiovascular complications were exclusive to males, while thrombo-embolic phenomena, arrhythmias, bone-related injuries, and procedural or dosing errors were unique to females. More information is included in Supplementary Tables S4, S5.

Age subgroup analysis indicated that neurotoxicity, allergic reactions, and QT interval monitoring are paramount in pediatric populations. In patients aged 18–65 years, close vigilance is required for ischemic heart disease progression and off-label medication risks. For patients older than 65 years, necessitate careful balancing of therapeutic efficacy against drug accumulation toxicity, with special emphasis on cognitive status and cardiac function dynamics. Age-related adverse event signal values are presented in Supplementary Tables S5-S7.

### 3.5 Time to onset and weibull distribution analysis of adverse events

**FIGURE 3:**
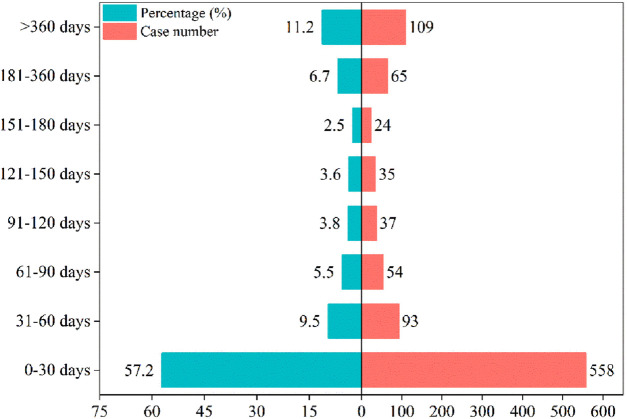
Time to onset of ranolazine-induced adverse events.

**FIGURE 4:**
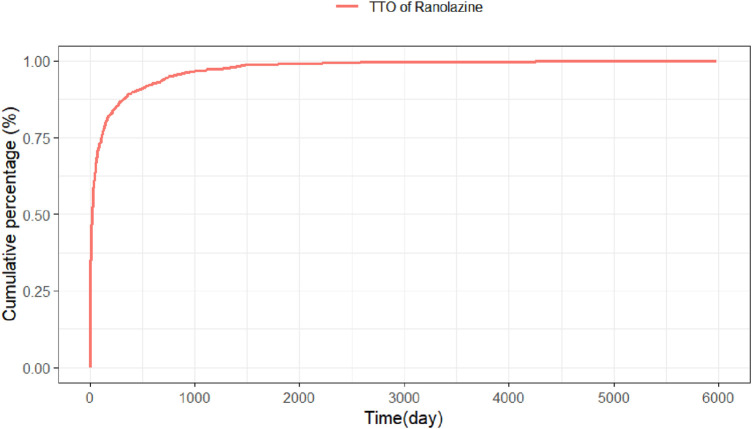
Cumulative incidence of ranolazine-induced adverse events over time. TTO, time to onset.

Adverse event beginning timings were collected for 975 patients, and the precise distribution of these occurrences is displayed in Figure 3. In terms of onset timing, ranolazine -related side effects mostly happened within the first month of therapy. The shape parameter (β) of the Weibull distribution was less than 1 (β = 0.49), indicating a decreasing hazard rate over time, which aligns with the observed early failure pattern where the risk of AEs is highest initially and decreases thereafter. Furthermore, the cumulative incidence curve of adverse events can be observed in Figure 4. Weibull distribution analysis revealed an early failure mode. More detailed parameters are outlined in Table 4.

**TABLE 4:** Time to onset of ranolazine -associated adverse events and weibull distribution analysis.

Drug	TTO (days)		Weibull distribution		
	Case reports	Median(d) (IQR)	Scale parameter: α(95%CI)	Shape parameter: β(95%CI)	Type
Ranolazine	975	20 (106)	65.09 (56.30, 73.88)	0.49 (0.47, 0.52)	Early failure

Abbreviation: TTO, time to onset; CI, confidence interval; IQR, interquartile range.

### 3.6 Sensitivity analysis

Ranolazine is commonly used in combination with metoprolol, amlodipine, aspirin, isosorbide mononitrate and atorvastatin. Following the removal of the five medications that are most frequently used in conjunction with ranolazine in clinical settings, PTs with more than 100 cases were reanalyzed. The potential adverse reactions at the PT level were discovered to be generally in line with previous investigations, in accordance with Supplementary Table S8, including dizziness, nausea, asthenia, constipation, headache, palpitations, chest pain, cardiac disorder, electrocardiogram qt prolonged, hypertension, seizure and myocardial infarction. This consistency strengthened the hypothesis that these signals may be associated with ranolazine itself, though confounding by other unmeasured factors or concomitant medications not excluded in this analysis cannot be entirely ruled out.​​

## 4 Discussion

This research evaluated ranolazine adverse events in detail when it was introduced to the market in 2006. By analyzing FAERS data, this study validated previously noted adverse reactions contained in the ranolazine medication label, including dizziness, nausea, asthenia, constipation, headache, palpitations, tinnitus, abdominal pain, dry mouth, vomiting, peripheral edema, dyspnea and hypotension. Moreover, adverse events not included on the label were discovered, such as cardiovascular events (e.g., chest pain, angina pectoris, atrial fibrillation, myocardial infarction), neurological disorders (e.g., seizure, tremor, myoclonus), and other vascular disorders (e.g., hypertension, pulmonary embolism). Cases of cardiac disorder and coronary artery disease were also reported. These results highlight the necessity of medication monitoring to efficiently control and minimize any negative effects, especially during the first month after starting therapy.

Numerous clinical studies report that dizziness and nausea are the most frequently reported AEs of ranolazine. For instance, A non-interventional prospective study to evaluate the efficacy and safety of ranolazine (Olympios et al., 2024) reported that the most common adverse reactions were dizziness and nausea. Further, a 4-week open-label dose-ascending study of ranolazine in amyotrophic lateral sclerosis (ALS) (Chandrashekhar et al., 2022) detected higher frequency of gastrointestinal AEs compared to known side effects of ranolazine (40% vs. 5%). Our results align with those of earlier studies. It's critical to pay close attention to and manage such adverse effects when taking ranolazine.

Another adverse reaction warranting attention is hypotension. In line with our findings, Hammond et al. (2015) found that the incidence of hypotension was higher for the ranolazine group than the non-ranolazine group. In that research, a greater number of patients in the ranolazine group experienced severe hypotension within 3 days following surgery, and one patient’s ranolazine was stopped due to symptomatic hypotension. Koskinas et al. (2014) and Tsanaxidis et al. (2017) reported that patients in the amiodarone plus ranolazine had hypotension more frequently than those in the amiodarone-only group. Thus, physicians should be aware of the potential for hypotension while administering ranolazine.

The emergence of significant signals for neurological adverse events, including seizure, myoclonus, and tremor, constituted a novel and intriguing aspect of our findings. Unlike the well-documented dizziness, these specific events are not currently featured in the drug’s label. Its plausible biological pathway exists, as pharmacokinetic studies (Jerling, 2006) confirm that ranolazine crosses the blood-brain barrier. The mechanism, while not fully elucidated, may involve off-target modulation of central nervous system (CNS) ion channels (e.g., sodium channels) or neurotransmitter systems (Sossalla et al., 2010), potentially altering neuronal excitability and lowering the seizure threshold. A plausible mechanistic pathway involves the off-target modulation of CNS-specific ion channels or neurotransmitter systems: Ranolazine’s primary pharmacological action is the inhibition of cardiac late sodium current (INaL), but it also exhibits weak affinity for voltage-gated sodium channels (VGSCs) expressed in neurons (e.g., Nav1.1, which is critical for GABAergic interneuron excitability) (de Lera Ruiz and Kraus, 2015). Additionally, recent *in vitro* studies have suggested that ranolazine may alter GABAergic neurotransmission by reducing GABA reuptake transporters (GAT-1), further enhancing neuronal excitability (Kuga et al., 2024). Although the absolute number of reports was low, the strength of the disproportionality signals warrants serious consideration. These results generated a new hypothesis that ranolazine may exert previously unrecognized effects on the CNS, urging clinicians to be vigilant for the emergence of such neurological symptoms during treatment.

The detection of a significant signal for atrial fibrillation (AF) presented a complex yet pharmacologically plausible finding. While ranolazine was indicated for chronic stable angina, its electrophysiological properties—namely the inhibition of the late sodium current (INaL) and a minor suppression of the rapid delayed rectifier potassium current (IKr)—theoretically confer a dual potential for both anti-arrhythmic and pro-arrhythmic effects (Antzelevitch et al., 2004). The propensity to prolong action potential duration and the QT interval, albeit modestly (Gong et al., 2017; Leelapatana et al., 2021), could create a substrate conducive to the development of re-entrant arrhythmias like AF in a vulnerable subset of patients. This signal, previously anecdotally reported, was substantiated here by a large-scale data-driven approach, necessitating a heightened clinical awareness.

The signal detected for pulmonary embolism (PE) was among the most unexpected findings of our study, as no direct pharmacological mechanism linking ranolazine to hypercoagulability or venous thromboembolism was currently known. While this association could represent a chance finding or heightened reporting bias due to the event’s severity, an indirect causal pathway merited speculative consideration. One could speculate that an indirect pathway might exist, whereby known adverse effects of ranolazine, such as asthenia, dizziness, or hypotension, may theoretically lead to reduced mobility (Heit, 2015). This, in turn, might predispose susceptible individuals to venous stasis and deep vein thrombosis, potentially culminating in PE. This hypothesis aligned with real-world reports of asthenia and syncope clustering within the first month of therapy—the same window in which PE events were observed. Alternative explanations cannot be excluded. Ranolazine has been shown to inhibit late sodium currents in platelets *in vitro*, an effect that might theoretically enhance platelet activation thresholds (Sharp et al., 2021). Platelet activation is a crucial step in thrombosis formation. The sodium ion influx mediated by sodium channels is involved in the processes of platelet degranulation, adhesion and aggregation (Evlakhov et al., 2024). However, existing studies have not found a direct effect of ranolazine on platelet function. Conversely, off-target modulation of ion channels in megakaryocytes or endothelial cells could plausibly influence coagulation homeostasis. Nonetheless, this remained a hypothesis, and the signal should be interpreted with utmost caution. It underscored the necessity for this potential association to be rigorously investigated in studies designed to control for relevant confounders, such as immobility and comorbidities, before any clinical implications can be inferred.

The subgroup analysis emphasized that severe neurological and cardiovascular complications severe neurological and cardiovascular complications should be given more attention in males, potentially reflecting pharmacokinetic or hormonally mediated susceptibilities. In contrast, thromboembolic events and arrhythmias were uniquely observed in females, possibly linked to sex-specific risk factors or prescribing patterns. Furthermore, age stratification identified distinct monitoring priorities: neurotoxicity and QT prolongation in pediatric populations, disease progression and off-label use risks in adults (18–65 years), and heightened vulnerability to drug accumulation toxicity requiring careful balance of efficacy against cognitive and cardiac risks in older adults (>65 years). Additionally, our results demonstrated the majority of instances happened within the first month of administering ranolazine, and that these occurrences gradually decreased. This emphasizes how important it is to continually monitor for adverse events during the first month of therapy. Moreover, we discovered persistent possible side effects, such as dizziness, nausea, chest pain, cardiac disorder, electrocardiogram qt prolonged, hypertension, seizure and myocardial infarction linked to ranolazine monotherapy by using sensitivity analysis, suggesting they may be more directly associated with ranolazine itself rather than its typical drug combinations.

We must recognize the limitations of our research. Firstly, as a spontaneous reporting system, the FAERS database is subject to well-known limitations including under-reporting, over-reporting, and reporting biases (e.g., the Weber effect, notoriety bias), which can distort the apparent frequency and strength of drug-event associations. Secondly, numerous unquantifiable confounding factors that might influence adverse events, including patient comorbidities, concomitant medications, and detailed demographic data, were not adjusted for in our disproportionality analysis. While our sensitivity analysis attempted to address concomitant medications, residual confounding remains a significant limitation. Nonetheless, our study improved the specificity of the results by concentrating on the drug itself as well as its precise usage indications. Thirdly, reporting bias may limit the generalizability of our findings to other ethnic or healthcare populations with different prescribing practices, genetic backgrounds, or comorbidity profiles due to the fact that most of the data came from the US. The goal of future studies should be to improve generalizability by combining data from several nations. Lastly, despite its ability to detect positive indicators of adverse events, disproportionality analysis failed to prove a causal association between ranolazine and these occurrences. It is imperative to conduct more prospective research to confirm these results and improve our comprehension of the safety profile of ranolazine.

## 5 Conclusion

In our study, we carried out a thorough analysis of ranolazine-related adverse events utilizing the FAERS database, concentrating on reports received since 2006. The research both validated adverse reactions mentioned on the medication’s label and potential adverse advents including chest pain, cardiac disorder, electrocardiogram qt prolonged, hypertension, seizure, tremor, atrial fibrillation, coronary artery disease, pulmonary embolism, myoclonus and myocardial infarction. Clinicians should do comprehensive pre-treatment evaluations and keep routine monitoring during the therapy, while patients should be made aware of potential side effects and encouraged to report problems as soon as possible.

## Data Availability

Publicly available datasets were analyzed in this study. This data can be found here: https://fis.fda.gov/extensions/FPD-QDE-FAERS/FPD-QDE-FAERS.html.
