# Supplementary material for: The real-world safety profile of ranolazine: pharmacovigilance analysis of the FAERS database
Source: Front Pharmacol. 2025 Nov 10;16:1702875. doi: 10.3389/fphar.2025.1702875 (PMC12640984; doi:10.3389/fphar.2025.1702875)
Supplement: Supplementary file 1 [file Table1.docx]

Supplementary Material

Supplementary Tables

Supplementary Table 1:

Two-by-two contingency table for disproportionality analyses.

|  | Target AEs | Other AEs | Total |
| --- | --- | --- | --- |
|  | a | b | a+b |
| Other drugs | c | d | c+d |
| Total | a+c | b+d | a+b+c+d |

Abbreviation: AEs, adverse events; a, number of reports containing both the target drug and target adverse drug reaction; b, number of reports containing other adverse drug reaction of the target drug; c, number of reports containing the target adverse drug reaction of other drugs; d, number of reports containing other drugs and other adverse drug reactions.

Supplementary Table 2:

Four major algorithms used for signal detection.

| Algorithms | Equation | Criteria |
| --- | --- | --- |
| ROR | ROR=ad/b/c | lower limit of 95% CI>1, N≥3 |
|  | 95%CI=e^ln(ROR)±1.96(1/a+1/b+1/c+1/d)^0.5^ |  |
| PRR | PRR=a(c+d)/c/(a+b) | PRR≥2, χ^2^≥4, N≥3 |
|  | χ^2^=[(ad-bc)^2](a+b+c+d)/[(a+b)(c+d)(a+c)(b+d)] |  |
| BCPNN | IC=log_2_a(a+b+c+d)(a+c)(a+b) | IC025>0 |
|  | 95%CI= E(IC) ± 2V(IC)^0.5 |  |
| MGPS | EBGM=a(a+b+c+d)/(a+c)/(a+b) | EBGM05>2 |
|  | 95%CI=e^ln(EBGM)±1.96(1/a+1/b+1/c+1/d)^0.5^ |  |

Abbreviation: a, number of reports containing both the target drug and target adverse drug reaction; b, number of reports containing other adverse drug reaction of the target drug; c, number of reports containing the target adverse drug reaction of other drugs; d, number of reports containing other drugs and other adverse drug reactions. 95%CI, 95% confidence interval; N, the number of reports; χ2, chi-squared; IC, information component; IC025, the lower limit of 95% CI of the IC; E(IC), the IC expectations; V(IC), the variance of IC; EBGM, empirical Bayesian geometric mean; EBGM05, the lower limit of 95% CI of EBGM.

Supplementary Table 3:

All adverse events meeting the positive signal threshold at the PT level from FAERS data

| PT | Case numbers | ROR(95%CI) | PRR(χ^2^) | EBGM(EBGM05) | IC(IC025) |
| --- | --- | --- | --- | --- | --- |
| Myocardial infarction | 660 | 8.04 ( 7.44 - 8.69 ) | 7.88 ( 3961.37 ) | 7.85 ( 7.27 ) | 2.97 ( 2.84 ) |
| Chest pain | 639 | 7.53 ( 6.96 - 8.15 ) | 7.39 ( 3527.42 ) | 7.36 ( 6.81 ) | 2.88 ( 2.75 ) |
| Angina pectoris | 589 | 44.92 ( 41.36 - 48.78 ) | 44.01 ( 24242.33 ) | 43.1 ( 39.68 ) | 5.43 ( 5.21 ) |
| Stent placement | 587 | 174.01 ( 159.83 - 189.46 ) | 170.47 ( 91193.14 ) | 157.25 ( 144.43 ) | 7.3 ( 6.83 ) |
| Dizziness | 583 | 2.57 ( 2.37 - 2.79 ) | 2.54 ( 548.29 ) | 2.54 ( 2.34 ) | 1.34 ( 1.22 ) |
| Cerebrovascular accident | 377 | 4.78 ( 4.32 - 5.3 ) | 4.73 ( 1110.59 ) | 4.72 ( 4.27 ) | 2.24 ( 2.08 ) |
| Intentional product use issue | 293 | 7.49 ( 6.68 - 8.41 ) | 7.43 ( 1625.58 ) | 7.4 ( 6.6 ) | 2.89 ( 2.69 ) |
| Constipation | 268 | 2.78 ( 2.46 - 3.13 ) | 2.76 ( 301.32 ) | 2.76 ( 2.44 ) | 1.46 ( 1.28 ) |
| Cardiac disorder | 260 | 5.97 ( 5.28 - 6.75 ) | 5.93 ( 1063.44 ) | 5.91 ( 5.23 ) | 2.56 ( 2.36 ) |
| Diabetes mellitus | 254 | 7.15 ( 6.31 - 8.09 ) | 7.09 ( 1326.01 ) | 7.07 ( 6.25 ) | 2.82 ( 2.61 ) |
| Seizure | 229 | 2.88 ( 2.53 - 3.28 ) | 2.86 ( 278.21 ) | 2.86 ( 2.51 ) | 1.52 ( 1.31 ) |
| Hypotension | 221 | 2.4 ( 2.1 - 2.74 ) | 2.39 ( 178.28 ) | 2.38 ( 2.09 ) | 1.25 ( 1.05 ) |
| Hypoacusis | 208 | 9.3 ( 8.11 - 10.66 ) | 9.24 ( 1521.84 ) | 9.2 ( 8.02 ) | 3.2 ( 2.95 ) |
| Product use issue | 203 | 2.43 ( 2.11 - 2.79 ) | 2.42 ( 169.07 ) | 2.42 ( 2.1 ) | 1.27 ( 1.06 ) |
| Cardiac failure congestive | 196 | 5.04 ( 4.38 - 5.8 ) | 5.01 ( 629.03 ) | 5 ( 4.35 ) | 2.32 ( 2.09 ) |
| Cardiac operation | 193 | 52.19 ( 45.22 - 60.24 ) | 51.85 ( 9384.47 ) | 50.57 ( 43.82 ) | 5.66 ( 5.12 ) |
| Cardiac arrest | 150 | 3.89 ( 3.31 - 4.57 ) | 3.87 ( 319.48 ) | 3.87 ( 3.29 ) | 1.95 ( 1.69 ) |
| Syncope | 145 | 3.1 ( 2.63 - 3.65 ) | 3.09 ( 204.52 ) | 3.08 ( 2.62 ) | 1.62 ( 1.36 ) |
| Unevaluable event | 143 | 4.04 ( 3.42 - 4.76 ) | 4.02 ( 324.18 ) | 4.01 ( 3.41 ) | 2.01 ( 1.73 ) |
| Cardiac failure | 131 | 3.53 ( 2.97 - 4.19 ) | 3.52 ( 235.97 ) | 3.51 ( 2.96 ) | 1.81 ( 1.53 ) |
| Atrial fibrillation | 127 | 2.81 ( 2.36 - 3.35 ) | 2.8 ( 147.5 ) | 2.8 ( 2.35 ) | 1.49 ( 1.21 ) |
| Coronary artery disease | 122 | 8.24 ( 6.9 - 9.85 ) | 8.21 ( 770.23 ) | 8.18 ( 6.85 ) | 3.03 ( 2.69 ) |
| Pulmonary embolism | 116 | 2.6 ( 2.16 - 3.11 ) | 2.59 ( 113.14 ) | 2.59 ( 2.16 ) | 1.37 ( 1.08 ) |
| Cardiac pacemaker insertion | 115 | 42.14 ( 35.02 - 50.7 ) | 41.97 ( 4506.16 ) | 41.14 ( 34.19 ) | 5.36 ( 4.66 ) |
| Dementia | 112 | 8.97 ( 7.45 - 10.81 ) | 8.94 ( 787.01 ) | 8.91 ( 7.4 ) | 3.16 ( 2.78 ) |
| Myoclonus | 106 | 19.3 ( 15.93 - 23.38 ) | 19.23 ( 1815.17 ) | 19.06 ( 15.74 ) | 4.25 ( 3.75 ) |
| Acute myocardial infarction | 104 | 7.42 ( 6.12 - 9 ) | 7.4 ( 573.42 ) | 7.37 ( 6.08 ) | 2.88 ( 2.51 ) |
| Catheterisation cardiac | 103 | 50.75 ( 41.72 - 61.73 ) | 50.57 ( 4882.52 ) | 49.36 ( 40.58 ) | 5.63 ( 4.79 ) |
| Coronary artery bypass | 101 | 36.5 ( 29.97 - 44.45 ) | 36.38 ( 3413.41 ) | 35.75 ( 29.35 ) | 5.16 ( 4.45 ) |
| Neoplasm malignant | 101 | 3.28 ( 2.7 - 3.99 ) | 3.27 ( 159.49 ) | 3.27 ( 2.69 ) | 1.71 ( 1.39 ) |
| Generalised tonic-clonic seizure | 98 | 8.22 ( 6.74 - 10.03 ) | 8.2 ( 617.25 ) | 8.17 ( 6.7 ) | 3.03 ( 2.64 ) |
| Chromaturia | 96 | 8.99 ( 7.35 - 10.99 ) | 8.96 ( 676.15 ) | 8.93 ( 7.3 ) | 3.16 ( 2.75 ) |
| Vascular graft | 96 | 69.11 ( 56.37 - 84.72 ) | 68.88 ( 6209.79 ) | 66.63 ( 54.35 ) | 6.06 ( 5.01 ) |
| Coronary arterial stent insertion | 95 | 41.04 ( 33.49 - 50.3 ) | 40.91 ( 3625.57 ) | 40.12 ( 32.73 ) | 5.33 ( 4.53 ) |
| Thrombosis | 94 | 2.51 ( 2.05 - 3.07 ) | 2.5 ( 84.71 ) | 2.5 ( 2.04 ) | 1.32 ( 1 ) |
| Electrocardiogram qt prolonged | 93 | 5.56 ( 4.53 - 6.82 ) | 5.54 ( 345.58 ) | 5.53 ( 4.51 ) | 2.47 ( 2.1 ) |
| Wrong product administered | 91 | 12.53 ( 10.19 - 15.4 ) | 12.49 ( 956.49 ) | 12.42 ( 10.11 ) | 3.63 ( 3.16 ) |
| Coronary artery occlusion | 90 | 13.78 ( 11.2 - 16.96 ) | 13.74 ( 1056.04 ) | 13.65 ( 11.09 ) | 3.77 ( 3.28 ) |
| Incorrect route of product administration | 83 | 3.33 ( 2.68 - 4.13 ) | 3.32 ( 134.58 ) | 3.32 ( 2.67 ) | 1.73 ( 1.38 ) |
| Deafness | 82 | 6.87 ( 5.53 - 8.54 ) | 6.85 ( 408.79 ) | 6.83 ( 5.5 ) | 2.77 ( 2.36 ) |
| Intentional dose omission | 82 | 7.74 ( 6.23 - 9.62 ) | 7.72 ( 478.24 ) | 7.7 ( 6.19 ) | 2.94 ( 2.51 ) |
| Ventricular tachycardia | 77 | 10.06 ( 8.04 - 12.59 ) | 10.04 ( 623.65 ) | 9.99 ( 7.99 ) | 3.32 ( 2.84 ) |
| Chronic obstructive pulmonary disease | 71 | 2.91 ( 2.3 - 3.67 ) | 2.9 ( 88.58 ) | 2.9 ( 2.3 ) | 1.54 ( 1.16 ) |
| Surgery | 70 | 2.79 ( 2.21 - 3.53 ) | 2.79 ( 80.29 ) | 2.79 ( 2.2 ) | 1.48 ( 1.1 ) |
| Implantable defibrillator insertion | 69 | 117.04 ( 91.79 - 149.23 ) | 116.76 ( 7485.62 ) | 110.42 ( 86.6 ) | 6.79 ( 5.07 ) |
| Anaphylactic reaction | 67 | 2.72 ( 2.14 - 3.45 ) | 2.71 ( 72.49 ) | 2.71 ( 2.13 ) | 1.44 ( 1.05 ) |
| Bradycardia | 66 | 2.65 ( 2.08 - 3.38 ) | 2.65 ( 67.63 ) | 2.65 ( 2.08 ) | 1.4 ( 1.02 ) |
| Arterial occlusive disease | 63 | 17.16 ( 13.39 - 22 ) | 17.13 ( 948.81 ) | 16.99 ( 13.26 ) | 4.09 ( 3.4 ) |
| Status epilepticus | 62 | 12.04 ( 9.38 - 15.46 ) | 12.02 ( 622.63 ) | 11.95 ( 9.31 ) | 3.58 ( 2.98 ) |
| Blindness | 59 | 3.18 ( 2.46 - 4.11 ) | 3.18 ( 87.83 ) | 3.17 ( 2.46 ) | 1.67 ( 1.24 ) |
| Disability | 50 | 5.52 ( 4.18 - 7.29 ) | 5.51 ( 184.34 ) | 5.5 ( 4.17 ) | 2.46 ( 1.93 ) |
| Product use complaint | 48 | 6.92 ( 5.21 - 9.19 ) | 6.91 ( 241.76 ) | 6.89 ( 5.19 ) | 2.78 ( 2.21 ) |
| Parkinson's disease | 48 | 5.52 ( 4.16 - 7.33 ) | 5.51 ( 176.81 ) | 5.5 ( 4.14 ) | 2.46 ( 1.92 ) |
| Product residue present | 46 | 7.74 ( 5.79 - 10.34 ) | 7.73 ( 268.62 ) | 7.71 ( 5.77 ) | 2.95 ( 2.33 ) |
| Transient ischaemic attack | 44 | 2.76 ( 2.05 - 3.71 ) | 2.76 ( 49.22 ) | 2.75 ( 2.05 ) | 1.46 ( 0.98 ) |
| Hip fracture | 43 | 2.92 ( 2.17 - 3.95 ) | 2.92 ( 54.31 ) | 2.92 ( 2.16 ) | 1.55 ( 1.05 ) |
| Dialysis | 42 | 6.33 ( 4.67 - 8.57 ) | 6.32 ( 187.61 ) | 6.31 ( 4.66 ) | 2.66 ( 2.05 ) |
| Ventricular fibrillation | 41 | 7.91 ( 5.82 - 10.75 ) | 7.9 ( 246.19 ) | 7.87 ( 5.79 ) | 2.98 ( 2.31 ) |
| Torsade de pointes | 41 | 11.34 ( 8.34 - 15.42 ) | 11.33 ( 383.99 ) | 11.27 ( 8.29 ) | 3.49 ( 2.73 ) |
| Angioplasty | 36 | 52.19 ( 37.48 - 72.67 ) | 52.12 ( 1759.64 ) | 50.83 ( 36.51 ) | 5.67 ( 3.96 ) |
| Circulatory collapse | 36 | 4.49 ( 3.24 - 6.23 ) | 4.49 ( 97.31 ) | 4.48 ( 3.23 ) | 2.16 ( 1.56 ) |
| Dementia alzheimer's type | 35 | 8.06 ( 5.78 - 11.23 ) | 8.05 ( 215.29 ) | 8.02 ( 5.76 ) | 3 ( 2.26 ) |
| Ischaemic stroke | 33 | 3.98 ( 2.83 - 5.6 ) | 3.98 ( 73.36 ) | 3.97 ( 2.82 ) | 1.99 ( 1.37 ) |
| Hip arthroplasty | 33 | 4.86 ( 3.45 - 6.84 ) | 4.86 ( 100.84 ) | 4.85 ( 3.44 ) | 2.28 ( 1.63 ) |
| Hallucination, visual | 30 | 3.17 ( 2.22 - 4.54 ) | 3.17 ( 44.5 ) | 3.17 ( 2.21 ) | 1.66 ( 1.05 ) |
| Knee arthroplasty | 30 | 3.23 ( 2.26 - 4.62 ) | 3.23 ( 46.11 ) | 3.23 ( 2.25 ) | 1.69 ( 1.07 ) |
| Glaucoma | 29 | 3.25 ( 2.26 - 4.68 ) | 3.25 ( 44.99 ) | 3.24 ( 2.25 ) | 1.7 ( 1.07 ) |
| Spinal operation | 27 | 5.19 ( 3.56 - 7.57 ) | 5.18 ( 90.96 ) | 5.17 ( 3.55 ) | 2.37 ( 1.62 ) |
| Blood pressure abnormal | 27 | 3.02 ( 2.07 - 4.41 ) | 3.02 ( 36.49 ) | 3.02 ( 2.07 ) | 1.59 ( 0.95 ) |
| Macular degeneration | 27 | 4.95 ( 3.39 - 7.22 ) | 4.95 ( 84.82 ) | 4.94 ( 3.38 ) | 2.3 ( 1.57 ) |
| Inability to afford medication | 27 | 6.27 ( 4.3 - 9.15 ) | 6.26 ( 119.09 ) | 6.25 ( 4.28 ) | 2.64 ( 1.85 ) |
| Neurotoxicity | 26 | 3.37 ( 2.29 - 4.94 ) | 3.36 ( 43.11 ) | 3.36 ( 2.29 ) | 1.75 ( 1.07 ) |
| Haemodynamic instability | 26 | 7.8 ( 5.31 - 11.47 ) | 7.8 ( 153.47 ) | 7.77 ( 5.29 ) | 2.96 ( 2.08 ) |
| Coronary artery thrombosis | 25 | 26.1 ( 17.59 - 38.73 ) | 26.08 ( 595.26 ) | 25.76 ( 17.36 ) | 4.69 ( 3.15 ) |
| Blindness unilateral | 24 | 3.71 ( 2.48 - 5.53 ) | 3.7 ( 47.28 ) | 3.7 ( 2.48 ) | 1.89 ( 1.16 ) |
| Product packaging confusion | 24 | 21.53 ( 14.4 - 32.2 ) | 21.52 ( 464.58 ) | 21.3 ( 14.24 ) | 4.41 ( 2.97 ) |
| Intentional underdose | 23 | 6.97 ( 4.63 - 10.5 ) | 6.96 ( 117.09 ) | 6.94 ( 4.61 ) | 2.8 ( 1.89 ) |
| Vasodilatation | 22 | 18.9 ( 12.42 - 28.76 ) | 18.88 ( 369.16 ) | 18.72 ( 12.3 ) | 4.23 ( 2.8 ) |
| Vascular stent thrombosis | 22 | 23.84 ( 15.66 - 36.31 ) | 23.83 ( 475.47 ) | 23.56 ( 15.47 ) | 4.56 ( 2.97 ) |
| Myoclonic epilepsy | 21 | 32.63 ( 21.2 - 50.23 ) | 32.61 ( 633.15 ) | 32.1 ( 20.86 ) | 5 ( 3.11 ) |
| Ventricular extrasystoles | 21 | 4.27 ( 2.78 - 6.55 ) | 4.26 ( 52.34 ) | 4.26 ( 2.77 ) | 2.09 ( 1.27 ) |
| Retinal artery occlusion | 20 | 17.47 ( 11.25 - 27.13 ) | 17.46 ( 307.63 ) | 17.32 ( 11.15 ) | 4.11 ( 2.65 ) |
| Cardiac ventricular thrombosis | 19 | 9.33 ( 5.94 - 14.64 ) | 9.32 ( 140.51 ) | 9.28 ( 5.91 ) | 3.21 ( 2.07 ) |
| Knee operation | 18 | 4.22 ( 2.66 - 6.7 ) | 4.22 ( 44.09 ) | 4.21 ( 2.65 ) | 2.07 ( 1.19 ) |
| Myocardial ischaemia | 18 | 3.25 ( 2.05 - 5.17 ) | 3.25 ( 28.03 ) | 3.25 ( 2.05 ) | 1.7 ( 0.88 ) |
| Angina unstable | 17 | 5.3 ( 3.29 - 8.54 ) | 5.3 ( 59.15 ) | 5.29 ( 3.28 ) | 2.4 ( 1.41 ) |
| Hip surgery | 17 | 7.3 ( 4.53 - 11.75 ) | 7.29 ( 91.96 ) | 7.27 ( 4.51 ) | 2.86 ( 1.75 ) |
| Atrioventricular block | 17 | 4.74 ( 2.94 - 7.62 ) | 4.73 ( 49.96 ) | 4.73 ( 2.94 ) | 2.24 ( 1.29 ) |
| Cardiac failure chronic | 17 | 8.75 ( 5.43 - 14.09 ) | 8.74 ( 116.07 ) | 8.71 ( 5.41 ) | 3.12 ( 1.93 ) |
| Heart valve replacement | 17 | 25.58 ( 15.85 - 41.28 ) | 25.57 ( 396.31 ) | 25.26 ( 15.65 ) | 4.66 ( 2.74 ) |
| Pulseless electrical activity | 17 | 7.49 ( 4.65 - 12.05 ) | 7.48 ( 95.13 ) | 7.46 ( 4.63 ) | 2.9 ( 1.77 ) |
| Aortic valve replacement | 15 | 24.37 ( 14.65 - 40.56 ) | 24.36 ( 332.01 ) | 24.08 ( 14.47 ) | 4.59 ( 2.57 ) |
| Myopathy | 14 | 3.45 ( 2.04 - 5.83 ) | 3.45 ( 24.33 ) | 3.45 ( 2.04 ) | 1.79 ( 0.82 ) |
| Walking aid user | 14 | 4.61 ( 2.73 - 7.78 ) | 4.6 ( 39.42 ) | 4.6 ( 2.72 ) | 2.2 ( 1.14 ) |
| Ischaemia | 14 | 6.38 ( 3.77 - 10.78 ) | 6.37 ( 63.25 ) | 6.36 ( 3.76 ) | 2.67 ( 1.48 ) |
| Aneurysm | 13 | 5.65 ( 3.28 - 9.74 ) | 5.65 ( 49.59 ) | 5.63 ( 3.27 ) | 2.49 ( 1.31 ) |
| Dysgraphia | 13 | 4.19 ( 2.43 - 7.22 ) | 4.19 ( 31.48 ) | 4.18 ( 2.43 ) | 2.06 ( 1 ) |
| Stress cardiomyopathy | 13 | 5.04 ( 2.92 - 8.69 ) | 5.04 ( 41.97 ) | 5.03 ( 2.92 ) | 2.33 ( 1.19 ) |
| Ischaemic cardiomyopathy | 13 | 8.59 ( 4.98 - 14.81 ) | 8.59 ( 86.79 ) | 8.56 ( 4.96 ) | 3.1 ( 1.7 ) |
| Haemoglobin increased | 13 | 6.61 ( 3.84 - 11.4 ) | 6.61 ( 61.69 ) | 6.59 ( 3.82 ) | 2.72 ( 1.46 ) |
| Arteriosclerosis coronary artery | 13 | 3.86 ( 2.24 - 6.65 ) | 3.86 ( 27.48 ) | 3.85 ( 2.24 ) | 1.95 ( 0.91 ) |
| Atrioventricular block first degree | 13 | 6.23 ( 3.62 - 10.75 ) | 6.23 ( 56.91 ) | 6.21 ( 3.61 ) | 2.64 ( 1.41 ) |
| Atrioventricular block complete | 13 | 4.13 ( 2.4 - 7.11 ) | 4.13 ( 30.74 ) | 4.12 ( 2.39 ) | 2.04 ( 0.98 ) |
| Cardiac assistance device user | 13 | 75.54 ( 43.42 - 131.43 ) | 75.5 ( 921.23 ) | 72.81 ( 41.85 ) | 6.19 ( 2.78 ) |
| Electrocardiogram abnormal | 13 | 3.49 ( 2.03 - 6.02 ) | 3.49 ( 23.07 ) | 3.49 ( 2.02 ) | 1.8 ( 0.79 ) |
| Cardioversion | 12 | 18.25 ( 10.33 - 32.21 ) | 18.24 ( 193.78 ) | 18.08 ( 10.24 ) | 4.18 ( 2.16 ) |
| Leg amputation | 12 | 6.16 ( 3.5 - 10.86 ) | 6.16 ( 51.73 ) | 6.15 ( 3.49 ) | 2.62 ( 1.34 ) |
| Product size issue | 12 | 5.01 ( 2.84 - 8.82 ) | 5 ( 38.36 ) | 4.99 ( 2.83 ) | 2.32 ( 1.13 ) |
| Arteriospasm coronary | 12 | 6.74 ( 3.82 - 11.88 ) | 6.73 ( 58.41 ) | 6.72 ( 3.81 ) | 2.75 ( 1.42 ) |
| Carotid artery occlusion | 12 | 7.03 ( 3.99 - 12.39 ) | 7.03 ( 61.81 ) | 7.01 ( 3.97 ) | 2.81 ( 1.46 ) |
| Arterial disorder | 12 | 13.01 ( 7.38 - 22.96 ) | 13.01 ( 132.19 ) | 12.93 ( 7.33 ) | 3.69 ( 1.95 ) |
| Catheter placement | 12 | 13.94 ( 7.9 - 24.61 ) | 13.94 ( 143.15 ) | 13.85 ( 7.85 ) | 3.79 ( 2 ) |
| Urine odour abnormal | 12 | 4.37 ( 2.48 - 7.7 ) | 4.37 ( 31.11 ) | 4.36 ( 2.48 ) | 2.12 ( 0.99 ) |
| Limb operation | 11 | 5.3 ( 2.93 - 9.58 ) | 5.3 ( 38.29 ) | 5.29 ( 2.93 ) | 2.4 ( 1.13 ) |
| Eye operation | 11 | 5.7 ( 3.15 - 10.3 ) | 5.7 ( 42.49 ) | 5.68 ( 3.14 ) | 2.51 ( 1.2 ) |
| Angiopathy | 10 | 3.76 ( 2.02 - 7 ) | 3.76 ( 20.25 ) | 3.76 ( 2.02 ) | 1.91 ( 0.72 ) |
| Myopathy toxic | 10 | 39.15 ( 20.94 - 73.2 ) | 39.13 ( 364.52 ) | 38.41 ( 20.54 ) | 5.26 ( 2.25 ) |
| Brash syndrome | 10 | 17.65 ( 9.47 - 32.9 ) | 17.65 ( 155.69 ) | 17.5 ( 9.39 ) | 4.13 ( 1.93 ) |
| Heart rate abnormal | 10 | 4.01 ( 2.15 - 7.45 ) | 4.01 ( 22.51 ) | 4 ( 2.15 ) | 2 ( 0.78 ) |
| Arterial thrombosis | 10 | 11.08 ( 5.95 - 20.64 ) | 11.08 ( 91.2 ) | 11.02 ( 5.92 ) | 3.46 ( 1.65 ) |
| Sudden cardiac death | 10 | 6.04 ( 3.25 - 11.23 ) | 6.04 ( 41.89 ) | 6.02 ( 3.24 ) | 2.59 ( 1.18 ) |
| Vascular occlusion | 10 | 10.13 ( 5.44 - 18.85 ) | 10.12 ( 81.81 ) | 10.08 ( 5.41 ) | 3.33 ( 1.59 ) |
| Product appearance confusion | 10 | 24.89 ( 13.34 - 46.44 ) | 24.88 ( 226.42 ) | 24.59 ( 13.18 ) | 4.62 ( 2.09 ) |
| Maternal exposure during delivery | 10 | 26.42 ( 14.16 - 49.31 ) | 26.41 ( 241.36 ) | 26.09 ( 13.98 ) | 4.71 ( 2.11 ) |
| Clonus | 9 | 7.13 ( 3.71 - 13.72 ) | 7.13 ( 47.27 ) | 7.11 ( 3.69 ) | 2.83 ( 1.23 ) |
| Cardiac ablation | 9 | 11.62 ( 6.04 - 22.38 ) | 11.62 ( 86.85 ) | 11.56 ( 6 ) | 3.53 ( 1.58 ) |
| Atrial thrombosis | 9 | 9.18 ( 4.77 - 17.67 ) | 9.17 ( 65.26 ) | 9.14 ( 4.75 ) | 3.19 ( 1.42 ) |
| Venous thrombosis | 9 | 4.73 ( 2.46 - 9.09 ) | 4.73 ( 26.38 ) | 4.72 ( 2.45 ) | 2.24 ( 0.87 ) |
| Vascular stent stenosis | 9 | 25.38 ( 13.15 - 48.98 ) | 25.37 ( 208.06 ) | 25.07 ( 12.99 ) | 4.65 ( 1.96 ) |
| Renal cortical necrosis | 9 | 88.51 ( 45.4 - 172.56 ) | 88.48 ( 745.7 ) | 84.8 ( 43.5 ) | 6.41 ( 2.24 ) |
| Labile blood pressure | 9 | 16.16 ( 8.38 - 31.14 ) | 16.15 ( 126.91 ) | 16.03 ( 8.32 ) | 4 ( 1.76 ) |
| Peripheral artery thrombosis | 9 | 9.95 ( 5.17 - 19.15 ) | 9.94 ( 72.05 ) | 9.9 ( 5.14 ) | 3.31 ( 1.47 ) |
| Cervical vertebral fracture | 9 | 5.71 ( 2.97 - 10.99 ) | 5.71 ( 34.86 ) | 5.7 ( 2.96 ) | 2.51 ( 1.04 ) |
| Angiogram | 8 | 63.75 ( 31.53 - 128.87 ) | 63.73 ( 478.81 ) | 61.81 ( 30.57 ) | 5.95 ( 2.02 ) |
| Hyperreflexia | 8 | 4.84 ( 2.42 - 9.69 ) | 4.84 ( 24.33 ) | 4.83 ( 2.41 ) | 2.27 ( 0.8 ) |
| Ventricular arrhythmia | 8 | 4.69 ( 2.34 - 9.38 ) | 4.69 ( 23.14 ) | 4.68 ( 2.34 ) | 2.23 ( 0.77 ) |
| Arterial stent insertion | 8 | 63.49 ( 31.41 - 128.36 ) | 63.48 ( 476.92 ) | 61.57 ( 30.46 ) | 5.94 ( 2.01 ) |
| Coronary angioplasty | 8 | 38.04 ( 18.9 - 76.56 ) | 38.03 ( 283.08 ) | 37.34 ( 18.55 ) | 5.22 ( 1.92 ) |
| Gallbladder operation | 8 | 4.46 ( 2.23 - 8.92 ) | 4.46 ( 21.39 ) | 4.45 ( 2.22 ) | 2.15 ( 0.72 ) |
| Ventricular hypokinesia | 8 | 5.22 ( 2.61 - 10.45 ) | 5.22 ( 27.22 ) | 5.21 ( 2.6 ) | 2.38 ( 0.86 ) |
| Product package associated injury | 8 | 8.08 ( 4.03 - 16.18 ) | 8.08 ( 49.42 ) | 8.05 ( 4.02 ) | 3.01 ( 1.21 ) |
| Microvascular coronary artery disease | 8 | 93.22 ( 45.88 - 189.41 ) | 93.2 ( 697.42 ) | 89.12 ( 43.86 ) | 6.48 ( 2.06 ) |
| Peripheral arterial occlusive disease | 8 | 4.07 ( 2.03 - 8.15 ) | 4.07 ( 18.49 ) | 4.06 ( 2.03 ) | 2.02 ( 0.64 ) |
| Gastrointestinal tube insertion | 8 | 7.14 ( 3.57 - 14.3 ) | 7.14 ( 42.1 ) | 7.12 ( 3.56 ) | 2.83 ( 1.12 ) |
| Aortic stenosis | 7 | 4.28 ( 2.04 - 8.99 ) | 4.28 ( 17.58 ) | 4.28 ( 2.04 ) | 2.1 ( 0.58 ) |
| Nodal rhythm | 7 | 10.08 ( 4.8 - 21.18 ) | 10.08 ( 56.95 ) | 10.03 ( 4.77 ) | 3.33 ( 1.21 ) |
| Hypercapnia | 7 | 5.1 ( 2.43 - 10.7 ) | 5.09 ( 22.98 ) | 5.08 ( 2.42 ) | 2.35 ( 0.73 ) |
| Hypercoagulation | 7 | 7.74 ( 3.68 - 16.26 ) | 7.74 ( 40.92 ) | 7.71 ( 3.67 ) | 2.95 ( 1.05 ) |
| Chemotherapy | 7 | 4.57 ( 2.18 - 9.6 ) | 4.57 ( 19.48 ) | 4.56 ( 2.17 ) | 2.19 ( 0.64 ) |
| Tonic clonic movements | 7 | 11.55 ( 5.49 - 24.28 ) | 11.54 ( 67.04 ) | 11.48 ( 5.46 ) | 3.52 ( 1.29 ) |
| Vascular dementia | 7 | 17.64 ( 8.38 - 37.12 ) | 17.63 ( 108.89 ) | 17.49 ( 8.31 ) | 4.13 ( 1.49 ) |
| Urinary tract obstruction | 7 | 5.4 ( 2.57 - 11.34 ) | 5.4 ( 25.04 ) | 5.39 ( 2.57 ) | 2.43 ( 0.78 ) |
| Urinary bladder haemorrhage | 7 | 7.12 ( 3.39 - 14.96 ) | 7.12 ( 36.71 ) | 7.1 ( 3.38 ) | 2.83 ( 0.99 ) |
| Atrioventricular block second degree | 7 | 4.89 ( 2.33 - 10.26 ) | 4.88 ( 21.58 ) | 4.88 ( 2.32 ) | 2.29 ( 0.69 ) |
| Paresis | 6 | 5.66 ( 2.54 - 12.61 ) | 5.66 ( 22.95 ) | 5.65 ( 2.53 ) | 2.5 ( 0.67 ) |
| Life support | 6 | 21.99 ( 9.84 - 49.17 ) | 21.99 ( 118.89 ) | 21.76 ( 9.73 ) | 4.44 ( 1.36 ) |
| Neck surgery | 6 | 6 ( 2.69 - 13.38 ) | 6 ( 24.93 ) | 5.99 ( 2.69 ) | 2.58 ( 0.71 ) |
| Dropped head syndrome | 6 | 35.68 ( 15.91 - 79.98 ) | 35.67 ( 198.68 ) | 35.07 ( 15.64 ) | 5.13 ( 1.48 ) |
| Systemic infection | 6 | 7.67 ( 3.44 - 17.1 ) | 7.67 ( 34.66 ) | 7.64 ( 3.43 ) | 2.93 ( 0.88 ) |
| Cerebral thrombosis | 6 | 4.53 ( 2.04 - 10.1 ) | 4.53 ( 16.49 ) | 4.53 ( 2.03 ) | 2.18 ( 0.5 ) |
| Autonomic nervous system imbalance | 6 | 4.68 ( 2.1 - 10.44 ) | 4.68 ( 17.34 ) | 4.67 ( 2.1 ) | 2.22 ( 0.52 ) |
| Peripheral artery occlusion | 6 | 7.47 ( 3.35 - 16.64 ) | 7.46 ( 33.47 ) | 7.44 ( 3.34 ) | 2.9 ( 0.86 ) |
| Tetany | 5 | 5.83 ( 2.42 - 14.03 ) | 5.83 ( 19.96 ) | 5.82 ( 2.42 ) | 2.54 ( 0.51 ) |
| Fixed eruption | 5 | 6.09 ( 2.53 - 14.65 ) | 6.09 ( 21.2 ) | 6.07 ( 2.52 ) | 2.6 ( 0.54 ) |
| Perineal pain | 5 | 16.55 ( 6.86 - 39.91 ) | 16.55 ( 72.44 ) | 16.42 ( 6.81 ) | 4.04 ( 1.02 ) |
| Foot amputation | 5 | 7.27 ( 3.02 - 17.49 ) | 7.27 ( 26.92 ) | 7.24 ( 3.01 ) | 2.86 ( 0.65 ) |
| Pco2 abnormal | 5 | 223.97 ( 88.9 - 564.28 ) | 223.93 ( 998.72 ) | 201.64 ( 80.03 ) | 7.66 ( 1.3 ) |
| Microangiopathy | 5 | 11.41 ( 4.74 - 27.49 ) | 11.41 ( 47.23 ) | 11.35 ( 4.71 ) | 3.51 ( 0.88 ) |
| Cardiac fibrillation | 5 | 7.35 ( 3.05 - 17.68 ) | 7.34 ( 27.31 ) | 7.32 ( 3.04 ) | 2.87 ( 0.65 ) |
| Ureteric obstruction | 5 | 7.76 ( 3.23 - 18.69 ) | 7.76 ( 29.35 ) | 7.74 ( 3.21 ) | 2.95 ( 0.68 ) |
| Ureteric perforation | 5 | 265.23 ( 104.39 - 673.91 ) | 265.18 ( 1162.93 ) | 234.47 ( 92.28 ) | 7.87 ( 1.3 ) |
| Therapeutic procedure | 5 | 8.49 ( 3.53 - 20.44 ) | 8.49 ( 32.9 ) | 8.46 ( 3.51 ) | 3.08 ( 0.73 ) |
| Stent malfunction | 5 | 52.49 ( 21.6 - 127.57 ) | 52.48 ( 246.11 ) | 51.18 ( 21.06 ) | 5.68 ( 1.25 ) |
| Orthostatic intolerance | 5 | 15.16 ( 6.29 - 36.54 ) | 15.15 ( 65.6 ) | 15.05 ( 6.24 ) | 3.91 ( 0.99 ) |
| Ovarian vein thrombosis | 5 | 78.13 ( 31.97 - 190.91 ) | 78.12 ( 366.44 ) | 75.24 ( 30.79 ) | 6.23 ( 1.29 ) |
| Cauda equina syndrome | 5 | 13.92 ( 5.78 - 33.55 ) | 13.92 ( 59.54 ) | 13.83 ( 5.74 ) | 3.79 ( 0.96 ) |
| Drug dose titration not performed | 5 | 6.87 ( 2.86 - 16.54 ) | 6.87 ( 25.01 ) | 6.85 ( 2.85 ) | 2.78 ( 0.61 ) |
| False negative investigation result | 5 | 15.39 ( 6.38 - 37.1 ) | 15.38 ( 66.74 ) | 15.28 ( 6.34 ) | 3.93 ( 0.99 ) |
| Cardiac rehabilitation therapy | 5 | 141.95 ( 57.31 - 351.59 ) | 141.93 ( 653.65 ) | 132.66 ( 53.56 ) | 7.05 ( 1.31 ) |
| Ischaemic cerebral infarction | 5 | 9.38 ( 3.9 - 22.59 ) | 9.38 ( 37.27 ) | 9.34 ( 3.88 ) | 3.22 ( 0.78 ) |
| Eschar | 4 | 11.12 ( 4.16 - 29.71 ) | 11.12 ( 36.64 ) | 11.06 ( 4.14 ) | 3.47 ( 0.58 ) |
| Arterial repair | 4 | 40.93 ( 15.21 - 110.14 ) | 40.92 ( 152.69 ) | 40.13 ( 14.91 ) | 5.33 ( 0.88 ) |
| Cancer surgery | 4 | 8.26 ( 3.09 - 22.06 ) | 8.26 ( 25.42 ) | 8.23 ( 3.08 ) | 3.04 ( 0.46 ) |
| Colon operation | 4 | 7.75 ( 2.9 - 20.68 ) | 7.74 ( 23.4 ) | 7.72 ( 2.89 ) | 2.95 ( 0.43 ) |
| Pneumonectomy | 4 | 47.43 ( 17.6 - 127.83 ) | 47.42 ( 177.59 ) | 46.35 ( 17.2 ) | 5.53 ( 0.89 ) |
| Renal infarct | 4 | 5.91 ( 2.22 - 15.77 ) | 5.91 ( 16.27 ) | 5.9 ( 2.21 ) | 2.56 ( 0.28 ) |
| Fractured coccyx | 4 | 6.93 ( 2.6 - 18.5 ) | 6.93 ( 20.23 ) | 6.91 ( 2.59 ) | 2.79 ( 0.37 ) |
| Carotid artery disease | 4 | 6.47 ( 2.42 - 17.27 ) | 6.47 ( 18.44 ) | 6.45 ( 2.42 ) | 2.69 ( 0.33 ) |
| Seizure like phenomena | 4 | 5.4 ( 2.03 - 14.42 ) | 5.4 ( 14.32 ) | 5.39 ( 2.02 ) | 2.43 ( 0.23 ) |
| Electrocardiogram qt interval abnormal | 4 | 18.58 ( 6.94 - 49.73 ) | 18.58 ( 65.91 ) | 18.41 ( 6.88 ) | 4.2 ( 0.74 ) |
| Renal artery stent placement | 4 | 54.85 ( 20.31 - 148.1 ) | 54.84 ( 205.84 ) | 53.41 ( 19.78 ) | 5.74 ( 0.91 ) |
| Implantable cardiac monitor insertion | 4 | 92.67 ( 34.01 - 252.51 ) | 92.66 ( 346.75 ) | 88.63 ( 32.53 ) | 6.47 ( 0.93 ) |
| Cardiac stress test abnormal | 4 | 9.92 ( 3.71 - 26.49 ) | 9.92 ( 31.91 ) | 9.87 ( 3.7 ) | 3.3 ( 0.54 ) |
| Carotid bruit | 3 | 15.27 ( 4.9 - 47.55 ) | 15.27 ( 39.7 ) | 15.16 ( 4.87 ) | 3.92 ( 0.29 ) |
| Pneumocephalus | 3 | 20.43 ( 6.55 - 63.71 ) | 20.43 ( 54.87 ) | 20.23 ( 6.49 ) | 4.34 ( 0.35 ) |
| Cardiospasm | 3 | 12.27 ( 3.94 - 38.16 ) | 12.26 ( 30.85 ) | 12.2 ( 3.92 ) | 3.61 ( 0.23 ) |
| Arterial injury | 3 | 8.65 ( 2.78 - 26.89 ) | 8.65 ( 20.21 ) | 8.62 ( 2.77 ) | 3.11 ( 0.12 ) |
| Vasculitic rash | 3 | 8.29 ( 2.67 - 25.78 ) | 8.29 ( 19.16 ) | 8.26 ( 2.66 ) | 3.05 ( 0.11 ) |
| Carotid endarterectomy | 3 | 32.16 ( 10.28 - 100.64 ) | 32.16 ( 89.15 ) | 31.67 ( 10.12 ) | 4.99 ( 0.41 ) |
| Peripheral artery bypass | 3 | 49.56 ( 15.76 - 155.83 ) | 49.56 ( 139.31 ) | 48.39 ( 15.39 ) | 5.6 ( 0.45 ) |
| Cardiac stress test | 3 | 50.81 ( 16.16 - 159.81 ) | 50.81 ( 142.88 ) | 49.58 ( 15.77 ) | 5.63 ( 0.45 ) |
| Vascular stent occlusion | 3 | 16.08 ( 5.16 - 50.09 ) | 16.08 ( 42.09 ) | 15.96 ( 5.12 ) | 4 ( 0.3 ) |
| Diabetic gastroparesis | 3 | 17.18 ( 5.51 - 53.52 ) | 17.18 ( 45.32 ) | 17.04 ( 5.47 ) | 4.09 ( 0.31 ) |
| Dizziness exertional | 3 | 9.11 ( 2.93 - 28.31 ) | 9.11 ( 21.55 ) | 9.07 ( 2.92 ) | 3.18 ( 0.14 ) |
| Muscle discomfort | 3 | 9.25 ( 2.97 - 28.74 ) | 9.24 ( 21.96 ) | 9.21 ( 2.96 ) | 3.2 ( 0.15 ) |
| Nitrate compound therapy | 3 | 6046.84 ( 628.95 - 58135.6 ) | 6046.2 ( 4533.15 ) | 1512.3 ( 157.3 ) | 10.56 ( 0.06 ) |
| Cerebral artery thrombosis | 3 | 12.27 ( 3.94 - 38.16 ) | 12.26 ( 30.85 ) | 12.2 ( 3.92 ) | 3.61 ( 0.23 ) |
| Cardiac procedure complication | 3 | 10.18 ( 3.27 - 31.66 ) | 10.18 ( 24.71 ) | 10.13 ( 3.26 ) | 3.34 ( 0.18 ) |
| Keratolysis exfoliativa acquired | 3 | 262.91 ( 78.93 - 875.68 ) | 262.88 ( 692.34 ) | 232.66 ( 69.85 ) | 7.86 ( 0.43 ) |
| Superior sagittal sinus thrombosis | 3 | 7.82 ( 2.52 - 24.31 ) | 7.82 ( 17.78 ) | 7.8 ( 2.51 ) | 2.96 ( 0.08 ) |
| Product closure removal difficult | 3 | 7.26 ( 2.34 - 22.55 ) | 7.26 ( 16.13 ) | 7.24 ( 2.33 ) | 2.86 ( 0.05 ) |
| Ventricular tachyarrhythmia | 3 | 20.5 ( 6.57 - 63.93 ) | 20.5 ( 55.07 ) | 20.3 ( 6.51 ) | 4.34 ( 0.35 ) |
| Post procedural pulmonary embolism | 3 | 27.87 ( 8.92 - 87.08 ) | 27.86 ( 76.64 ) | 27.5 ( 8.8 ) | 4.78 ( 0.39 ) |
| Lipid metabolism disorder | 3 | 19.08 ( 6.12 - 59.47 ) | 19.07 ( 50.9 ) | 18.9 ( 6.06 ) | 4.24 ( 0.34 ) |
| External counterpulsation | 3 | 2015.61 ( 406.79 - 9987.14 ) | 2015.4 ( 3020.1 ) | 1008.2 ( 203.48 ) | 9.98 ( 0.19 ) |
| Hepatic cancer metastatic | 3 | 7.29 ( 2.34 - 22.64 ) | 7.28 ( 16.21 ) | 7.26 ( 2.34 ) | 2.86 ( 0.05 ) |

Abbreviation: Asterisks (*) indicate statistically significant signals in algorithm; ROR, reporting odds ratio; PRR, proportional reporting ratio; EBGM, empirical Bayesian geometric mean; EBGM05, the lower limit of the 95% CI of EBGM; IC, information component; IC025, the lower limit of the 95% CI of the IC; CI, confidence interval; PT,preferred term; AEs, adverse events.

Supplementary Table 4:

Top 50 most frequent adverse events for ranolazine at the PT level in males from FAERS data

| PT | Case numbers | ROR(95%CI) | PRR(χ^2^) | EBGM(EBGM05) | IC(IC025) |
| --- | --- | --- | --- | --- | --- |
| Myocardial infarction | 398 | 6.51 ( 5.89 - 7.19 ) | 6.35 ( 1794.23 ) | 6.33 ( 5.73 ) | 2.66 ( 2.5 ) |
| Stent placement | 345 | 135.1 ( 120.82 - 151.07 ) | 131.82 ( 40889.73 ) | 120.4 ( 107.68 ) | 6.91 ( 6.32 ) |
| Chest pain | 336 | 7.85 ( 7.04 - 8.75 ) | 7.69 ( 1949.2 ) | 7.65 ( 6.86 ) | 2.94 ( 2.75 ) |
| Angina pectoris | 308 | 35.79 ( 31.92 - 40.12 ) | 35.03 ( 9935.12 ) | 34.18 ( 30.49 ) | 5.1 ( 4.78 ) |
| Dizziness | 294 | 2.91 ( 2.59 - 3.27 ) | 2.87 ( 360.51 ) | 2.87 ( 2.55 ) | 1.52 ( 1.34 ) |
| Cerebrovascular accident | 217 | 4.67 ( 4.08 - 5.34 ) | 4.61 ( 613.68 ) | 4.6 ( 4.02 ) | 2.2 ( 1.98 ) |
| Cardiac disorder | 155 | 5.59 ( 4.77 - 6.55 ) | 5.54 ( 574.99 ) | 5.52 ( 4.71 ) | 2.46 ( 2.19 ) |
| Intentional product use issue | 154 | 8.46 ( 7.21 - 9.92 ) | 8.38 ( 995.94 ) | 8.33 ( 7.11 ) | 3.06 ( 2.76 ) |
| Constipation | 148 | 3.19 ( 2.71 - 3.75 ) | 3.16 ( 219.06 ) | 3.16 ( 2.68 ) | 1.66 ( 1.4 ) |
| Hypoacusis | 146 | 11.07 ( 9.4 - 13.04 ) | 10.97 ( 1313.37 ) | 10.89 ( 9.24 ) | 3.44 ( 3.11 ) |
| Diabetes mellitus | 137 | 6.48 ( 5.48 - 7.68 ) | 6.43 ( 626.39 ) | 6.41 ( 5.41 ) | 2.68 ( 2.38 ) |
| Cardiac failure congestive | 123 | 5.18 ( 4.33 - 6.19 ) | 5.14 ( 409.47 ) | 5.13 ( 4.29 ) | 2.36 ( 2.05 ) |
| Cardiac operation | 111 | 42.63 ( 35.27 - 51.54 ) | 42.31 ( 4344.22 ) | 41.08 ( 33.98 ) | 5.36 ( 4.64 ) |
| Product use issue | 97 | 2.51 ( 2.06 - 3.07 ) | 2.5 ( 87.4 ) | 2.5 ( 2.05 ) | 1.32 ( 1.01 ) |
| Unevaluable event | 87 | 4.89 ( 3.96 - 6.04 ) | 4.87 ( 266.92 ) | 4.86 ( 3.93 ) | 2.28 ( 1.91 ) |
| Cardiac failure | 77 | 3.16 ( 2.52 - 3.95 ) | 3.15 ( 112.71 ) | 3.14 ( 2.51 ) | 1.65 ( 1.28 ) |
| Coronary artery disease | 75 | 5.96 ( 4.75 - 7.49 ) | 5.94 ( 306.9 ) | 5.92 ( 4.71 ) | 2.56 ( 2.14 ) |
| Cardiac pacemaker insertion | 74 | 44.11 ( 34.98 - 55.63 ) | 43.88 ( 3005.84 ) | 42.56 ( 33.75 ) | 5.41 ( 4.44 ) |
| Dementia | 72 | 9.71 ( 7.7 - 12.26 ) | 9.67 ( 556.07 ) | 9.61 ( 7.62 ) | 3.26 ( 2.76 ) |
| Cardiac arrest | 70 | 2.61 ( 2.07 - 3.31 ) | 2.6 ( 69.2 ) | 2.6 ( 2.06 ) | 1.38 ( 1 ) |
| Coronary artery bypass | 69 | 27.55 ( 21.7 - 34.98 ) | 27.42 ( 1722.64 ) | 26.91 ( 21.19 ) | 4.75 ( 3.95 ) |
| Neoplasm malignant | 63 | 3.8 ( 2.97 - 4.88 ) | 3.79 ( 129.33 ) | 3.78 ( 2.95 ) | 1.92 ( 1.5 ) |
| Vascular graft | 62 | 56.24 ( 43.6 - 72.53 ) | 56 ( 3218.37 ) | 53.85 ( 41.75 ) | 5.75 ( 4.5 ) |
| Deafness | 61 | 9.47 ( 7.36 - 12.19 ) | 9.43 ( 456.87 ) | 9.37 ( 7.28 ) | 3.23 ( 2.68 ) |
| Implantable defibrillator insertion | 54 | 110.51 ( 83.72 - 145.88 ) | 110.09 ( 5405.98 ) | 102.02 ( 77.29 ) | 6.67 ( 4.76 ) |
| Chromaturia | 53 | 8.12 ( 6.19 - 10.64 ) | 8.09 ( 327.56 ) | 8.05 ( 6.14 ) | 3.01 ( 2.44 ) |
| Myoclonus | 53 | 15.21 ( 11.6 - 19.95 ) | 15.16 ( 693.41 ) | 15 ( 11.44 ) | 3.91 ( 3.18 ) |
| Catheterisation cardiac | 52 | 47.13 ( 35.73 - 62.17 ) | 46.96 ( 2262.35 ) | 45.45 ( 34.46 ) | 5.51 ( 4.22 ) |
| Acute myocardial infarction | 52 | 4.36 ( 3.32 - 5.72 ) | 4.34 ( 133.58 ) | 4.33 ( 3.3 ) | 2.12 ( 1.63 ) |
| Coronary artery occlusion | 50 | 9.14 ( 6.92 - 12.08 ) | 9.11 ( 358.84 ) | 9.06 ( 6.86 ) | 3.18 ( 2.56 ) |
| Coronary arterial stent insertion | 47 | 28.95 ( 21.67 - 38.66 ) | 28.85 ( 1237.98 ) | 28.28 ( 21.18 ) | 4.82 ( 3.75 ) |
| Generalised tonic-clonic seizure | 45 | 6.51 ( 4.85 - 8.73 ) | 6.49 ( 208.08 ) | 6.46 ( 4.82 ) | 2.69 ( 2.1 ) |
| Surgery | 43 | 3.93 ( 2.91 - 5.31 ) | 3.92 ( 93.46 ) | 3.91 ( 2.9 ) | 1.97 ( 1.44 ) |
| Ventricular tachycardia | 41 | 7.39 ( 5.43 - 10.04 ) | 7.37 ( 224.51 ) | 7.33 ( 5.39 ) | 2.87 ( 2.22 ) |
| Parkinson's disease | 39 | 5.79 ( 4.23 - 7.94 ) | 5.78 ( 153.58 ) | 5.76 ( 4.2 ) | 2.53 ( 1.91 ) |
| Intentional dose omission | 39 | 8.32 ( 6.07 - 11.41 ) | 8.3 ( 249.13 ) | 8.26 ( 6.03 ) | 3.05 ( 2.35 ) |
| Electrocardiogram qt prolonged | 37 | 4.37 ( 3.17 - 6.04 ) | 4.37 ( 95.76 ) | 4.35 ( 3.15 ) | 2.12 ( 1.53 ) |
| Arterial occlusive disease | 37 | 13.41 ( 9.7 - 18.54 ) | 13.38 ( 419.74 ) | 13.26 ( 9.59 ) | 3.73 ( 2.85 ) |
| Anaphylactic reaction | 34 | 3.11 ( 2.22 - 4.35 ) | 3.1 ( 48.38 ) | 3.1 ( 2.21 ) | 1.63 ( 1.06 ) |
| Dialysis | 33 | 7.28 ( 5.17 - 10.25 ) | 7.26 ( 177.31 ) | 7.23 ( 5.13 ) | 2.85 ( 2.11 ) |
| Angioplasty | 30 | 67.59 ( 46.83 - 97.55 ) | 67.45 ( 1872.37 ) | 64.35 ( 44.59 ) | 6.01 ( 3.87 ) |
| Wrong product administered | 30 | 8.34 ( 5.82 - 11.95 ) | 8.32 ( 192.22 ) | 8.28 ( 5.78 ) | 3.05 ( 2.22 ) |
| Disability | 29 | 5.67 ( 3.94 - 8.17 ) | 5.66 ( 110.88 ) | 5.64 ( 3.92 ) | 2.5 ( 1.76 ) |
| Blindness | 28 | 3 ( 2.07 - 4.35 ) | 3 ( 37.19 ) | 2.99 ( 2.06 ) | 1.58 ( 0.95 ) |
| Product residue present | 27 | 9.1 ( 6.23 - 13.29 ) | 9.08 ( 192.98 ) | 9.03 ( 6.18 ) | 3.17 ( 2.26 ) |
| Dementia alzheimer's type | 27 | 11.43 ( 7.82 - 16.7 ) | 11.41 ( 254.41 ) | 11.33 ( 7.75 ) | 3.5 ( 2.5 ) |
| Status epilepticus | 25 | 8.19 ( 5.53 - 12.14 ) | 8.18 ( 156.67 ) | 8.14 ( 5.49 ) | 3.02 ( 2.11 ) |
| Ventricular fibrillation | 23 | 5.97 ( 3.97 - 9 ) | 5.97 ( 94.7 ) | 5.94 ( 3.95 ) | 2.57 ( 1.71 ) |
| Product use complaint | 22 | 8.06 ( 5.3 - 12.27 ) | 8.05 ( 135.14 ) | 8.01 ( 5.27 ) | 3 ( 2.01 ) |
| Neurotoxicity | 19 | 4.11 ( 2.62 - 6.45 ) | 4.11 ( 44.52 ) | 4.1 ( 2.61 ) | 2.03 ( 1.18 ) |

Abbreviation: Asterisks (*) indicate statistically significant signals in algorithm; ROR, reporting odds ratio; PRR, proportional reporting ratio; EBGM, empirical Bayesian geometric mean; EBGM05, the lower limit of the 95% CI of EBGM; IC, information component; IC025, the lower limit of the 95% CI of the IC; CI, confidence interval; PT,preferred term; AEs, adverse events.

Supplementary Table 5:

Top 50 most frequent adverse events for ranolazine at the PT level in females from FAERS data

| PT | Case numbers | ROR(95%CI) | PRR(χ^2^) | EBGM(EBGM05) | IC(IC025) |
| --- | --- | --- | --- | --- | --- |
| Chest pain | 284 | 7.08 ( 6.3 - 7.97 ) | 6.95 ( 1446.83 ) | 6.93 ( 6.16 ) | 2.79 ( 2.59 ) |
| Angina pectoris | 270 | 54.99 ( 48.68 - 62.11 ) | 53.86 ( 13718.47 ) | 52.75 ( 46.7 ) | 5.72 ( 5.29 ) |
| Stent placement | 241 | 214.81 ( 188.13 - 245.27 ) | 210.83 ( 46431.34 ) | 194.56 ( 170.4 ) | 7.6 ( 6.56 ) |
| Myocardial infarction | 239 | 9.32 ( 8.2 - 10.6 ) | 9.17 ( 1736.62 ) | 9.14 ( 8.04 ) | 3.19 ( 2.96 ) |
| Cerebrovascular accident | 152 | 4.82 ( 4.11 - 5.66 ) | 4.77 ( 453.72 ) | 4.77 ( 4.06 ) | 2.25 ( 1.98 ) |
| Diabetes mellitus | 116 | 7.81 ( 6.5 - 9.38 ) | 7.75 ( 680.68 ) | 7.73 ( 6.44 ) | 2.95 ( 2.6 ) |
| Intentional product use issue | 111 | 8.07 ( 6.69 - 9.73 ) | 8.01 ( 679.42 ) | 7.99 ( 6.62 ) | 3 ( 2.64 ) |
| Constipation | 108 | 2.47 ( 2.04 - 2.99 ) | 2.46 ( 93.65 ) | 2.46 ( 2.03 ) | 1.3 ( 1 ) |
| Cardiac disorder | 104 | 6.27 ( 5.17 - 7.61 ) | 6.23 ( 456.27 ) | 6.22 ( 5.13 ) | 2.64 ( 2.28 ) |
| Product use issue | 101 | 2.72 ( 2.24 - 3.31 ) | 2.71 ( 109.25 ) | 2.71 ( 2.23 ) | 1.44 ( 1.13 ) |
| Seizure | 90 | 2.81 ( 2.29 - 3.46 ) | 2.8 ( 104.39 ) | 2.8 ( 2.27 ) | 1.49 ( 1.15 ) |
| Cardiac operation | 81 | 60.57 ( 48.56 - 75.55 ) | 60.2 ( 4605.24 ) | 58.81 ( 47.15 ) | 5.88 ( 4.78 ) |
| Pulmonary embolism | 80 | 3.95 ( 3.17 - 4.93 ) | 3.94 ( 175.22 ) | 3.93 ( 3.16 ) | 1.98 ( 1.6 ) |
| Cardiac failure congestive | 68 | 4.72 ( 3.72 - 5.99 ) | 4.7 ( 197.83 ) | 4.69 ( 3.7 ) | 2.23 ( 1.81 ) |
| Syncope | 66 | 3.2 ( 2.51 - 4.08 ) | 3.19 ( 99.24 ) | 3.19 ( 2.5 ) | 1.67 ( 1.27 ) |
| Cardiac arrest | 66 | 4.87 ( 3.83 - 6.21 ) | 4.85 ( 201.73 ) | 4.85 ( 3.8 ) | 2.28 ( 1.84 ) |
| Hypoacusis | 62 | 6.5 ( 5.06 - 8.35 ) | 6.47 ( 286.44 ) | 6.46 ( 5.03 ) | 2.69 ( 2.21 ) |
| Thrombosis | 55 | 3.38 ( 2.59 - 4.41 ) | 3.37 ( 91.71 ) | 3.37 ( 2.58 ) | 1.75 ( 1.31 ) |
| Unevaluable event | 53 | 3.39 ( 2.59 - 4.44 ) | 3.38 ( 88.86 ) | 3.38 ( 2.58 ) | 1.76 ( 1.3 ) |
| Atrial fibrillation | 53 | 3.23 ( 2.47 - 4.23 ) | 3.22 ( 81.26 ) | 3.22 ( 2.46 ) | 1.69 ( 1.24 ) |
| Catheterisation cardiac | 51 | 55.37 ( 41.93 - 73.11 ) | 55.15 ( 2653.59 ) | 53.99 ( 40.89 ) | 5.75 ( 4.34 ) |
| Generalised tonic-clonic seizure | 46 | 9.34 ( 6.99 - 12.48 ) | 9.31 ( 340.08 ) | 9.28 ( 6.94 ) | 3.21 ( 2.56 ) |
| Coronary arterial stent insertion | 46 | 72.43 ( 54 - 97.15 ) | 72.18 ( 3138.62 ) | 70.19 ( 52.33 ) | 6.13 ( 4.4 ) |
| Myoclonus | 45 | 21.63 ( 16.12 - 29.03 ) | 21.56 ( 875.02 ) | 21.39 ( 15.94 ) | 4.42 ( 3.46 ) |
| Electrocardiogram qt prolonged | 45 | 6.51 ( 4.86 - 8.73 ) | 6.49 ( 208.56 ) | 6.48 ( 4.83 ) | 2.7 ( 2.11 ) |
| Coronary artery disease | 44 | 10.3 ( 7.65 - 13.85 ) | 10.26 ( 366.54 ) | 10.23 ( 7.6 ) | 3.35 ( 2.65 ) |
| Intentional dose omission | 43 | 9.2 ( 6.82 - 12.42 ) | 9.18 ( 312.26 ) | 9.15 ( 6.78 ) | 3.19 ( 2.51 ) |
| Acute myocardial infarction | 42 | 10.82 ( 7.99 - 14.66 ) | 10.79 ( 371.45 ) | 10.74 ( 7.93 ) | 3.43 ( 2.69 ) |
| Cardiac pacemaker insertion | 41 | 37.28 ( 27.37 - 50.77 ) | 37.16 ( 1421.83 ) | 36.63 ( 26.9 ) | 5.2 ( 3.86 ) |
| Dementia | 40 | 7.8 ( 5.72 - 10.65 ) | 7.78 ( 235.74 ) | 7.76 ( 5.69 ) | 2.96 ( 2.28 ) |
| Cardiac failure | 40 | 3.02 ( 2.22 - 4.12 ) | 3.02 ( 53.94 ) | 3.02 ( 2.21 ) | 1.59 ( 1.07 ) |
| Coronary artery occlusion | 39 | 18.88 ( 13.77 - 25.88 ) | 18.82 ( 653.42 ) | 18.69 ( 13.63 ) | 4.22 ( 3.24 ) |
| Wrong product administered | 39 | 14.42 ( 10.52 - 19.76 ) | 14.38 ( 482.74 ) | 14.3 ( 10.43 ) | 3.84 ( 2.97 ) |
| Incorrect route of product administration | 39 | 3.63 ( 2.65 - 4.97 ) | 3.62 ( 73.88 ) | 3.62 ( 2.64 ) | 1.85 ( 1.31 ) |
| Chromaturia | 38 | 8.7 ( 6.32 - 11.96 ) | 8.67 ( 257.2 ) | 8.65 ( 6.29 ) | 3.11 ( 2.39 ) |
| Neoplasm malignant | 38 | 3.35 ( 2.44 - 4.61 ) | 3.34 ( 62.36 ) | 3.34 ( 2.43 ) | 1.74 ( 1.19 ) |
| Chronic obstructive pulmonary disease | 35 | 3.37 ( 2.42 - 4.7 ) | 3.36 ( 58.07 ) | 3.36 ( 2.41 ) | 1.75 ( 1.17 ) |
| Vascular graft | 33 | 92.33 ( 65.2 - 130.74 ) | 92.1 ( 2868.32 ) | 88.87 ( 62.76 ) | 6.47 ( 4.13 ) |
| Therapeutic response unexpected | 33 | 3.19 ( 2.27 - 4.49 ) | 3.18 ( 49.39 ) | 3.18 ( 2.26 ) | 1.67 ( 1.08 ) |
| Bradycardia | 32 | 3.57 ( 2.52 - 5.05 ) | 3.56 ( 58.96 ) | 3.56 ( 2.52 ) | 1.83 ( 1.22 ) |
| Coronary artery bypass | 32 | 48.05 ( 33.85 - 68.2 ) | 47.93 ( 1442.99 ) | 47.05 ( 33.15 ) | 5.56 ( 3.79 ) |
| Blindness | 30 | 3.69 ( 2.58 - 5.28 ) | 3.68 ( 58.51 ) | 3.68 ( 2.57 ) | 1.88 ( 1.24 ) |
| Status epilepticus | 29 | 14.35 ( 9.96 - 20.68 ) | 14.32 ( 357.23 ) | 14.24 ( 9.88 ) | 3.83 ( 2.78 ) |
| Hip fracture | 28 | 3.53 ( 2.44 - 5.12 ) | 3.52 ( 50.6 ) | 3.52 ( 2.43 ) | 1.82 ( 1.16 ) |
| Arterial occlusive disease | 25 | 19.04 ( 12.84 - 28.23 ) | 19.01 ( 423.33 ) | 18.87 ( 12.73 ) | 4.24 ( 2.91 ) |
| Ventricular tachycardia | 23 | 9.06 ( 6.01 - 13.65 ) | 9.04 ( 163.98 ) | 9.01 ( 5.98 ) | 3.17 ( 2.17 ) |
| Circulatory collapse | 23 | 7.32 ( 4.86 - 11.03 ) | 7.31 ( 124.98 ) | 7.29 ( 4.84 ) | 2.87 ( 1.94 ) |
| Deafness | 21 | 4.08 ( 2.66 - 6.26 ) | 4.08 ( 48.68 ) | 4.07 ( 2.65 ) | 2.03 ( 1.22 ) |
| Hip arthroplasty | 21 | 5.91 ( 3.85 - 9.07 ) | 5.9 ( 85.28 ) | 5.89 ( 3.84 ) | 2.56 ( 1.65 ) |
| Product use complaint | 21 | 6.65 ( 4.34 - 10.22 ) | 6.65 ( 100.48 ) | 6.63 ( 4.32 ) | 2.73 ( 1.78 ) |

Abbreviation: Asterisks (*) indicate statistically significant signals in algorithm; ROR, reporting odds ratio; PRR, proportional reporting ratio; EBGM, empirical Bayesian geometric mean; EBGM05, the lower limit of the 95% CI of EBGM; IC, information component; IC025, the lower limit of the 95% CI of the IC; CI, confidence interval; PT,preferred term; AEs, adverse events.

Supplementary Table 6:

Adverse events at the PT level for ranolazine in patients aged under 18 from FAERS data

| PT | Case numbers | ROR(95%CI) | PRR(χ^2^) | EBGM(EBGM05) | IC(IC025) |
| --- | --- | --- | --- | --- | --- |
| Anaphylactic reaction | 19 | 24.21 ( 15.29 - 38.32 ) | 23.36 ( 405.31 ) | 23.25 ( 14.69 ) | 4.54 ( 2.8 ) |
| Hypotension | 16 | 9.66 ( 5.87 - 15.9 ) | 9.39 ( 120.18 ) | 9.38 ( 5.7 ) | 3.23 ( 1.94 ) |
| Intentional overdose | 16 | 8.61 ( 5.23 - 14.17 ) | 8.38 ( 104.14 ) | 8.36 ( 5.08 ) | 3.06 ( 1.83 ) |
| Seizure | 14 | 3.56 ( 2.09 - 6.05 ) | 3.49 ( 25 ) | 3.48 ( 2.05 ) | 1.8 ( 0.82 ) |
| Cardiac arrest | 13 | 13.67 ( 7.88 - 23.72 ) | 13.35 ( 148.4 ) | 13.32 ( 7.67 ) | 3.74 ( 2.04 ) |
| Retinal artery occlusion | 9 | 612.25 ( 304.83 - 1229.67 ) | 601.61 ( 4811.47 ) | 536.48 ( 267.11 ) | 9.07 ( 2.32 ) |
| Drug interaction | 8 | 5.56 ( 2.76 - 11.17 ) | 5.48 ( 29.39 ) | 5.48 ( 2.72 ) | 2.45 ( 0.9 ) |
| Generalised tonic-clonic seizure | 8 | 11.27 ( 5.6 - 22.67 ) | 11.11 ( 73.53 ) | 11.09 ( 5.51 ) | 3.47 ( 1.41 ) |
| Tachycardia | 7 | 4.73 ( 2.24 - 9.98 ) | 4.68 ( 20.31 ) | 4.68 ( 2.22 ) | 2.23 ( 0.65 ) |
| Suicidal ideation | 7 | 5.22 ( 2.48 - 11.02 ) | 5.17 ( 23.56 ) | 5.16 ( 2.45 ) | 2.37 ( 0.73 ) |
| Acute kidney injury | 7 | 5.41 ( 2.56 - 11.41 ) | 5.35 ( 24.78 ) | 5.34 ( 2.53 ) | 2.42 ( 0.76 ) |
| Ventricular fibrillation | 7 | 63.12 ( 29.8 - 133.69 ) | 62.28 ( 416.89 ) | 61.51 ( 29.04 ) | 5.94 ( 1.81 ) |
| Electrocardiogram qt prolonged | 7 | 12.28 ( 5.82 - 25.91 ) | 12.13 ( 71.38 ) | 12.1 ( 5.73 ) | 3.6 ( 1.31 ) |
| Wheezing | 6 | 12.62 ( 5.64 - 28.24 ) | 12.48 ( 63.26 ) | 12.45 ( 5.56 ) | 3.64 ( 1.14 ) |
| Haemorrhage | 6 | 5.56 ( 2.49 - 12.45 ) | 5.51 ( 22.18 ) | 5.51 ( 2.46 ) | 2.46 ( 0.64 ) |
| Status epilepticus | 5 | 11.69 ( 4.84 - 28.23 ) | 11.58 ( 48.28 ) | 11.56 ( 4.79 ) | 3.53 ( 0.88 ) |
| Blindness unilateral | 5 | 116.15 ( 47.65 - 283.09 ) | 115.04 ( 552.42 ) | 112.44 ( 46.13 ) | 6.81 ( 1.32 ) |
| Venoocclusive liver disease | 5 | 19.67 ( 8.14 - 47.54 ) | 19.49 ( 87.41 ) | 19.42 ( 8.03 ) | 4.28 ( 1.07 ) |
| Vascular stent thrombosis | 5 | 1664.94 ( 602.87 - 4598.03 ) | 1648.85 ( 6175.72 ) | 1236.88 ( 447.87 ) | 10.27 ( 1.24 ) |
| Shock | 4 | 11.9 ( 4.45 - 31.88 ) | 11.82 ( 39.55 ) | 11.79 ( 4.4 ) | 3.56 ( 0.6 ) |
| Alopecia | 4 | 6.14 ( 2.3 - 16.44 ) | 6.1 ( 17.07 ) | 6.1 ( 2.28 ) | 2.61 ( 0.3 ) |
| Blindness | 4 | 19.99 ( 7.46 - 53.58 ) | 19.85 ( 71.33 ) | 19.77 ( 7.38 ) | 4.31 ( 0.76 ) |
| Renal failure | 4 | 6.17 ( 2.31 - 16.51 ) | 6.13 ( 17.17 ) | 6.12 ( 2.29 ) | 2.61 ( 0.3 ) |
| Acute myocardial infarction | 4 | 97.26 ( 36.02 - 262.62 ) | 96.52 ( 370.91 ) | 94.69 ( 35.07 ) | 6.57 ( 0.95 ) |
| Hemiparesis | 3 | 18.89 ( 6.06 - 58.88 ) | 18.78 ( 50.34 ) | 18.72 ( 6 ) | 4.23 ( 0.33 ) |
| Thrombosis | 3 | 12.94 ( 4.15 - 40.31 ) | 12.87 ( 32.77 ) | 12.84 ( 4.12 ) | 3.68 ( 0.25 ) |
| Rash maculo-papular | 3 | 9.08 ( 2.92 - 28.27 ) | 9.03 ( 21.4 ) | 9.02 ( 2.9 ) | 3.17 ( 0.13 ) |
| Pulmonary embolism | 3 | 8.17 ( 2.62 - 25.43 ) | 8.13 ( 18.73 ) | 8.12 ( 2.61 ) | 3.02 ( 0.1 ) |
| Mental status changes | 3 | 8.18 ( 2.63 - 25.46 ) | 8.14 ( 18.76 ) | 8.12 ( 2.61 ) | 3.02 ( 0.1 ) |
| Haemodynamic instability | 3 | 22.44 ( 7.19 - 69.98 ) | 22.32 ( 60.82 ) | 22.22 ( 7.12 ) | 4.47 ( 0.36 ) |

Abbreviation: Asterisks (*) indicate statistically significant signals in algorithm; ROR, reporting odds ratio; PRR, proportional reporting ratio; EBGM, empirical Bayesian geometric mean; EBGM05, the lower limit of the 95% CI of EBGM; IC, information component; IC025, the lower limit of the 95% CI of the IC; CI, confidence interval; PT, preferred term.

Supplementary Table 7:

Top 50 most frequent adverse events for ranolazine at the PT level in patients aged 18 to 65 from FAERS data

| PT | Case numbers | ROR(95%CI) | PRR(χ^2^) | EBGM(EBGM05) | IC(IC025) |
| --- | --- | --- | --- | --- | --- |
| Chest pain | 262 | 8.62 ( 7.62 - 9.75 ) | 8.38 ( 1704.49 ) | 8.36 ( 7.39 ) | 3.06 ( 2.84 ) |
| Myocardial infarction | 230 | 10.13 ( 8.88 - 11.55 ) | 9.88 ( 1833.66 ) | 9.85 ( 8.63 ) | 3.3 ( 3.05 ) |
| Angina pectoris | 195 | 49.08 ( 42.53 - 56.63 ) | 47.96 ( 8815.83 ) | 47.15 ( 40.86 ) | 5.56 ( 5.04 ) |
| Stent placement | 188 | 230.52 ( 198.35 - 267.91 ) | 225.4 ( 38793.12 ) | 208.24 ( 179.18 ) | 7.7 ( 6.41 ) |
| Cerebrovascular accident | 109 | 6.41 ( 5.3 - 7.74 ) | 6.34 ( 489.79 ) | 6.32 ( 5.23 ) | 2.66 ( 2.32 ) |
| Intentional product use issue | 106 | 11.11 ( 9.17 - 13.46 ) | 10.98 ( 959.1 ) | 10.94 ( 9.03 ) | 3.45 ( 3.04 ) |
| Cardiac disorder | 83 | 9.99 ( 8.05 - 12.41 ) | 9.91 ( 662.81 ) | 9.87 ( 7.95 ) | 3.3 ( 2.84 ) |
| Product use issue | 82 | 4.44 ( 3.57 - 5.52 ) | 4.41 ( 216.01 ) | 4.4 ( 3.54 ) | 2.14 ( 1.76 ) |
| Hypotension | 79 | 2.99 ( 2.4 - 3.73 ) | 2.97 ( 103.66 ) | 2.97 ( 2.38 ) | 1.57 ( 1.21 ) |
| Pulmonary embolism | 71 | 4.63 ( 3.67 - 5.85 ) | 4.6 ( 200.22 ) | 4.6 ( 3.64 ) | 2.2 ( 1.79 ) |
| Cardiac operation | 68 | 91.4 ( 71.71 - 116.49 ) | 90.67 ( 5836.54 ) | 87.78 ( 68.87 ) | 6.46 ( 4.93 ) |
| Diabetes mellitus | 68 | 6.35 ( 5 - 8.06 ) | 6.3 ( 303.05 ) | 6.29 ( 4.95 ) | 2.65 ( 2.2 ) |
| Cardiac arrest | 59 | 4.18 ( 3.23 - 5.4 ) | 4.15 ( 141.36 ) | 4.15 ( 3.21 ) | 2.05 ( 1.61 ) |
| Coronary artery disease | 44 | 8.76 ( 6.51 - 11.79 ) | 8.72 ( 300.13 ) | 8.7 ( 6.47 ) | 3.12 ( 2.46 ) |
| Acute myocardial infarction | 44 | 9.61 ( 7.14 - 12.93 ) | 9.57 ( 336.57 ) | 9.54 ( 7.09 ) | 3.25 ( 2.57 ) |
| Syncope | 43 | 3.16 ( 2.34 - 4.26 ) | 3.15 ( 63.07 ) | 3.15 ( 2.33 ) | 1.65 ( 1.15 ) |
| Thrombosis | 42 | 4.44 ( 3.27 - 6.01 ) | 4.42 ( 111.04 ) | 4.41 ( 3.26 ) | 2.14 ( 1.59 ) |
| Vascular graft | 41 | 124.36 ( 90.88 - 170.19 ) | 123.76 ( 4775.74 ) | 118.43 ( 86.54 ) | 6.89 ( 4.51 ) |
| Cardiac failure congestive | 40 | 4.33 ( 3.17 - 5.91 ) | 4.31 ( 101.69 ) | 4.31 ( 3.16 ) | 2.11 ( 1.54 ) |
| Coronary arterial stent insertion | 39 | 62.97 ( 45.81 - 86.56 ) | 62.69 ( 2314.24 ) | 61.3 ( 44.59 ) | 5.94 ( 4.15 ) |
| Incorrect route of product administration | 39 | 7.78 ( 5.68 - 10.66 ) | 7.75 ( 228.68 ) | 7.73 ( 5.64 ) | 2.95 ( 2.27 ) |
| Catheterisation cardiac | 38 | 67.97 ( 49.23 - 93.85 ) | 67.67 ( 2435.62 ) | 66.05 ( 47.84 ) | 6.05 ( 4.16 ) |
| Myoclonus | 36 | 19.54 ( 14.07 - 27.13 ) | 19.46 ( 625.94 ) | 19.33 ( 13.91 ) | 4.27 ( 3.21 ) |
| Coronary artery occlusion | 35 | 16.81 ( 12.05 - 23.46 ) | 16.75 ( 515.24 ) | 16.65 ( 11.94 ) | 4.06 ( 3.05 ) |
| Deep vein thrombosis | 34 | 3.25 ( 2.32 - 4.55 ) | 3.24 ( 52.55 ) | 3.23 ( 2.31 ) | 1.69 ( 1.11 ) |
| Wrong product administered | 34 | 24.76 ( 17.65 - 34.73 ) | 24.67 ( 765.25 ) | 24.45 ( 17.43 ) | 4.61 ( 3.38 ) |
| Intentional dose omission | 33 | 12.09 ( 8.58 - 17.03 ) | 12.05 ( 332.9 ) | 12 ( 8.52 ) | 3.58 ( 2.68 ) |
| Generalised tonic-clonic seizure | 32 | 6.83 ( 4.83 - 9.67 ) | 6.81 ( 158.33 ) | 6.8 ( 4.8 ) | 2.76 ( 2.03 ) |
| Coronary artery bypass | 30 | 37.34 ( 26.03 - 53.57 ) | 37.21 ( 1043.06 ) | 36.73 ( 25.6 ) | 5.2 ( 3.57 ) |
| Status epilepticus | 28 | 16.46 ( 11.34 - 23.88 ) | 16.41 ( 402.73 ) | 16.31 ( 11.24 ) | 4.03 ( 2.88 ) |
| Unevaluable event | 27 | 3.52 ( 2.42 - 5.14 ) | 3.52 ( 48.61 ) | 3.51 ( 2.41 ) | 1.81 ( 1.14 ) |
| Electrocardiogram qt prolonged | 26 | 4.68 ( 3.18 - 6.88 ) | 4.67 ( 74.82 ) | 4.66 ( 3.17 ) | 2.22 ( 1.48 ) |
| Bradycardia | 25 | 3.88 ( 2.62 - 5.75 ) | 3.88 ( 53.29 ) | 3.87 ( 2.61 ) | 1.95 ( 1.23 ) |
| Surgery | 23 | 3.79 ( 2.52 - 5.71 ) | 3.78 ( 47.09 ) | 3.78 ( 2.51 ) | 1.92 ( 1.17 ) |
| Disability | 23 | 12.98 ( 8.61 - 19.56 ) | 12.95 ( 252.41 ) | 12.89 ( 8.55 ) | 3.69 ( 2.52 ) |
| Arterial occlusive disease | 23 | 22.78 ( 15.11 - 34.36 ) | 22.72 ( 473.74 ) | 22.54 ( 14.95 ) | 4.49 ( 2.98 ) |
| Hypoxia | 21 | 4.18 ( 2.72 - 6.42 ) | 4.17 ( 50.61 ) | 4.17 ( 2.72 ) | 2.06 ( 1.25 ) |
| Neurotoxicity | 21 | 9.33 ( 6.07 - 14.32 ) | 9.31 ( 155.2 ) | 9.28 ( 6.04 ) | 3.21 ( 2.14 ) |
| Hypoacusis | 21 | 6.74 ( 4.39 - 10.35 ) | 6.73 ( 102.22 ) | 6.72 ( 4.37 ) | 2.75 ( 1.8 ) |
| Chronic obstructive pulmonary disease | 21 | 4.19 ( 2.73 - 6.44 ) | 4.19 ( 50.89 ) | 4.18 ( 2.72 ) | 2.06 ( 1.25 ) |
| Circulatory collapse | 20 | 8.03 ( 5.17 - 12.46 ) | 8.01 ( 122.38 ) | 7.99 ( 5.15 ) | 3 ( 1.95 ) |
| Chromaturia | 17 | 5.1 ( 3.16 - 8.2 ) | 5.09 ( 55.74 ) | 5.08 ( 3.15 ) | 2.34 ( 1.37 ) |
| Impaired work ability | 17 | 3.96 ( 2.46 - 6.37 ) | 3.95 ( 37.44 ) | 3.95 ( 2.45 ) | 1.98 ( 1.08 ) |
| Ventricular tachycardia | 17 | 6.43 ( 4 - 10.36 ) | 6.42 ( 77.69 ) | 6.41 ( 3.98 ) | 2.68 ( 1.62 ) |
| Deafness | 16 | 6.88 ( 4.21 - 11.24 ) | 6.87 ( 80.07 ) | 6.86 ( 4.2 ) | 2.78 ( 1.65 ) |
| Implantable defibrillator insertion | 16 | 106.98 ( 64.89 - 176.37 ) | 106.78 ( 1613.33 ) | 102.78 ( 62.34 ) | 6.68 ( 3.16 ) |
| Cardiac pacemaker insertion | 15 | 40.43 ( 24.27 - 67.34 ) | 40.36 ( 567.35 ) | 39.78 ( 23.88 ) | 5.31 ( 2.81 ) |
| Angioplasty | 14 | 83.92 ( 49.28 - 142.89 ) | 83.78 ( 1110.9 ) | 81.31 ( 47.75 ) | 6.35 ( 2.92 ) |
| Haemodynamic instability | 13 | 11.15 ( 6.47 - 19.24 ) | 11.14 ( 119.49 ) | 11.1 ( 6.43 ) | 3.47 ( 1.91 ) |
| Ventricular fibrillation | 13 | 6.9 ( 4 - 11.9 ) | 6.89 ( 65.35 ) | 6.88 ( 3.99 ) | 2.78 ( 1.5 ) |

Abbreviation: Asterisks (*) indicate statistically significant signals in algorithm; ROR, reporting odds ratio; PRR, proportional reporting ratio; EBGM, empirical Bayesian geometric mean; EBGM05, the lower limit of the 95% CI of EBGM; IC, information component; IC025, the lower limit of the 95% CI of the IC; CI, confidence interval; PT, preferred term.

Supplementary Table 8:

Top 50 most frequent adverse events for ranolazine at the PT level in patients aged over 65 from FAERS data

| PT | Case numbers | ROR(95%CI) | PRR(χ^2^) | EBGM(EBGM05) | IC(IC025) |
| --- | --- | --- | --- | --- | --- |
| Stent placement | 348 | 139.97 ( 124.89 - 156.88 ) | 136.57 ( 40627.58 ) | 118.58 ( 105.8 ) | 6.89 ( 6.3 ) |
| Myocardial infarction | 333 | 6.7 ( 6.01 - 7.47 ) | 6.56 ( 1564.65 ) | 6.52 ( 5.85 ) | 2.71 ( 2.52 ) |
| Angina pectoris | 321 | 35.46 ( 31.68 - 39.69 ) | 34.68 ( 10114.8 ) | 33.42 ( 29.86 ) | 5.06 ( 4.76 ) |
| Chest pain | 274 | 6.4 ( 5.68 - 7.22 ) | 6.3 ( 1216.74 ) | 6.26 ( 5.55 ) | 2.65 ( 2.44 ) |
| Cerebrovascular accident | 215 | 3.84 ( 3.35 - 4.39 ) | 3.8 ( 442.75 ) | 3.78 ( 3.31 ) | 1.92 ( 1.7 ) |
| Hypoacusis | 156 | 8.88 ( 7.58 - 10.41 ) | 8.8 ( 1069 ) | 8.72 ( 7.44 ) | 3.12 ( 2.82 ) |
| Diabetes mellitus | 154 | 10.05 ( 8.57 - 11.8 ) | 9.96 ( 1228.46 ) | 9.86 ( 8.4 ) | 3.3 ( 2.99 ) |
| Cardiac disorder | 152 | 5.85 ( 4.98 - 6.86 ) | 5.79 ( 600.28 ) | 5.76 ( 4.91 ) | 2.53 ( 2.25 ) |
| Intentional product use issue | 147 | 8.34 ( 7.09 - 9.82 ) | 8.27 ( 931.62 ) | 8.2 ( 6.97 ) | 3.04 ( 2.73 ) |
| Cardiac failure congestive | 136 | 4.38 ( 3.7 - 5.19 ) | 4.35 ( 349.4 ) | 4.33 ( 3.66 ) | 2.11 ( 1.83 ) |
| Cardiac operation | 107 | 43.47 ( 35.78 - 52.81 ) | 43.15 ( 4203 ) | 41.2 ( 33.91 ) | 5.36 ( 4.62 ) |
| Product use issue | 103 | 2.67 ( 2.2 - 3.25 ) | 2.66 ( 106.73 ) | 2.66 ( 2.19 ) | 1.41 ( 1.1 ) |
| Dementia | 92 | 7.15 ( 5.82 - 8.78 ) | 7.11 ( 479.27 ) | 7.06 ( 5.74 ) | 2.82 ( 2.43 ) |
| Cardiac pacemaker insertion | 92 | 32.22 ( 26.16 - 39.7 ) | 32.02 ( 2669.76 ) | 30.95 ( 25.12 ) | 4.95 ( 4.24 ) |
| Memory impairment | 89 | 2.99 ( 2.43 - 3.68 ) | 2.98 ( 116.66 ) | 2.97 ( 2.41 ) | 1.57 ( 1.23 ) |
| Unevaluable event | 71 | 4.95 ( 3.92 - 6.25 ) | 4.93 ( 221.48 ) | 4.91 ( 3.89 ) | 2.3 ( 1.88 ) |
| Neoplasm malignant | 70 | 4.77 ( 3.77 - 6.04 ) | 4.75 ( 206.55 ) | 4.73 ( 3.74 ) | 2.24 ( 1.82 ) |
| Seizure | 69 | 3.17 ( 2.5 - 4.01 ) | 3.16 ( 101.49 ) | 3.15 ( 2.49 ) | 1.66 ( 1.26 ) |
| Coronary artery disease | 60 | 5.76 ( 4.47 - 7.43 ) | 5.74 ( 233.65 ) | 5.71 ( 4.43 ) | 2.51 ( 2.03 ) |
| Catheterisation cardiac | 60 | 47.64 ( 36.73 - 61.8 ) | 47.45 ( 2590.68 ) | 45.1 ( 34.77 ) | 5.5 ( 4.33 ) |
| Coronary artery bypass | 57 | 29.65 ( 22.76 - 38.62 ) | 29.54 ( 1521.34 ) | 28.62 ( 21.97 ) | 4.84 ( 3.89 ) |
| Myoclonus | 54 | 15.35 ( 11.72 - 20.1 ) | 15.3 ( 709.51 ) | 15.05 ( 11.5 ) | 3.91 ( 3.19 ) |
| Deafness | 51 | 7.26 ( 5.51 - 9.57 ) | 7.24 ( 272.22 ) | 7.19 ( 5.46 ) | 2.85 ( 2.28 ) |
| Generalised tonic-clonic seizure | 51 | 16.64 ( 12.61 - 21.96 ) | 16.58 ( 733.28 ) | 16.3 ( 12.35 ) | 4.03 ( 3.25 ) |
| Implantable defibrillator insertion | 48 | 111.98 ( 82.91 - 151.23 ) | 111.6 ( 4676.81 ) | 99.31 ( 73.53 ) | 6.63 ( 4.61 ) |
| Coronary artery occlusion | 48 | 10.43 ( 7.84 - 13.87 ) | 10.4 ( 403.21 ) | 10.29 ( 7.74 ) | 3.36 ( 2.7 ) |
| Chromaturia | 44 | 7.06 ( 5.25 - 9.51 ) | 7.05 ( 226.54 ) | 7 ( 5.2 ) | 2.81 ( 2.19 ) |
| Electrocardiogram qt prolonged | 44 | 4.65 ( 3.46 - 6.26 ) | 4.64 ( 125.17 ) | 4.62 ( 3.44 ) | 2.21 ( 1.67 ) |
| Acute myocardial infarction | 43 | 3.59 ( 2.66 - 4.85 ) | 3.58 ( 79.83 ) | 3.57 ( 2.65 ) | 1.84 ( 1.32 ) |
| Coronary arterial stent insertion | 43 | 30.29 ( 22.34 - 41.07 ) | 30.2 ( 1174.62 ) | 29.25 ( 21.57 ) | 4.87 ( 3.71 ) |
| Vascular graft | 42 | 44.4 ( 32.56 - 60.55 ) | 44.27 ( 1692.53 ) | 42.23 ( 30.96 ) | 5.4 ( 3.98 ) |
| Surgery | 38 | 4.04 ( 2.93 - 5.55 ) | 4.03 ( 86.18 ) | 4.01 ( 2.92 ) | 2.01 ( 1.43 ) |
| Amnesia | 38 | 2.84 ( 2.07 - 3.91 ) | 2.84 ( 45.16 ) | 2.83 ( 2.06 ) | 1.5 ( 0.97 ) |
| Intentional dose omission | 37 | 8.04 ( 5.82 - 11.12 ) | 8.02 ( 225.54 ) | 7.96 ( 5.76 ) | 2.99 ( 2.28 ) |
| Blindness | 32 | 3.66 ( 2.59 - 5.19 ) | 3.66 ( 61.61 ) | 3.65 ( 2.58 ) | 1.87 ( 1.25 ) |
| Ventricular tachycardia | 31 | 5.83 ( 4.1 - 8.31 ) | 5.82 ( 123.08 ) | 5.79 ( 4.07 ) | 2.53 ( 1.82 ) |
| Arterial occlusive disease | 31 | 11.35 ( 7.96 - 16.18 ) | 11.33 ( 288.33 ) | 11.2 ( 7.86 ) | 3.49 ( 2.57 ) |
| Wrong product administered | 29 | 9.03 ( 6.26 - 13.03 ) | 9.02 ( 204.63 ) | 8.93 ( 6.2 ) | 3.16 ( 2.29 ) |
| Dementia alzheimer's type | 29 | 6.51 ( 4.52 - 9.39 ) | 6.5 ( 134.13 ) | 6.46 ( 4.48 ) | 2.69 ( 1.92 ) |
| Parkinson's disease | 28 | 3.48 ( 2.4 - 5.04 ) | 3.47 ( 49.09 ) | 3.46 ( 2.39 ) | 1.79 ( 1.14 ) |
| Dialysis | 26 | 6.29 ( 4.28 - 9.25 ) | 6.28 ( 114.66 ) | 6.24 ( 4.24 ) | 2.64 ( 1.83 ) |
| Macular degeneration | 25 | 4.9 ( 3.31 - 7.26 ) | 4.89 ( 77.03 ) | 4.87 ( 3.29 ) | 2.28 ( 1.52 ) |
| Hip arthroplasty | 24 | 5.74 ( 3.84 - 8.58 ) | 5.73 ( 93.16 ) | 5.7 ( 3.81 ) | 2.51 ( 1.68 ) |
| Glaucoma | 23 | 3.74 ( 2.48 - 5.63 ) | 3.73 ( 45.85 ) | 3.72 ( 2.47 ) | 1.9 ( 1.15 ) |
| Hallucination, visual | 23 | 3.15 ( 2.09 - 4.75 ) | 3.15 ( 33.59 ) | 3.14 ( 2.08 ) | 1.65 ( 0.94 ) |
| Knee arthroplasty | 23 | 3.7 ( 2.46 - 5.58 ) | 3.7 ( 45.09 ) | 3.69 ( 2.45 ) | 1.88 ( 1.14 ) |
| Disability | 19 | 13.85 ( 8.8 - 21.8 ) | 13.84 ( 222.83 ) | 13.64 ( 8.67 ) | 3.77 ( 2.41 ) |
| Product residue present | 19 | 8.56 ( 5.44 - 13.45 ) | 8.55 ( 125.41 ) | 8.47 ( 5.39 ) | 3.08 ( 1.98 ) |
| Status epilepticus | 19 | 9.4 ( 5.98 - 14.78 ) | 9.39 ( 141.02 ) | 9.31 ( 5.92 ) | 3.22 ( 2.07 ) |
| Torsade de pointes | 19 | 6.85 ( 4.36 - 10.75 ) | 6.84 ( 93.99 ) | 6.79 ( 4.32 ) | 2.76 ( 1.75 ) |

Abbreviation: Asterisks (*) indicate statistically significant signals in algorithm; ROR, reporting odds ratio; PRR, proportional reporting ratio; EBGM, empirical Bayesian geometric mean; EBGM05, the lower limit of the 95% CI of EBGM; IC, information component; IC025, the lower limit of the 95% CI of the IC; CI, confidence interval; PT, preferred term.

Supplementary Table 9:

The frequent adverse events for ranolazine excluding common medication co-usage at the PT level from FAERS data

| PT | Case numbers | ROR(95%CI) | PRR(χ2) | EBGM(EBGM05) | IC(IC025) |
| --- | --- | --- | --- | --- | --- |
| Death* | 753 | 2.27 ( 2.11 - 2.44 ) | 2.23 ( 518.79 ) | 2.23 ( 2.1 ) | 1.16 ( 1.05 ) |
| Myocardial infarction* | 623 | 9.34 ( 8.63 - 10.12 ) | 9.12 ( 4499.53 ) | 9.09 ( 8.5 ) | 3.18 ( 3.07 ) |
| Chest pain* | 575 | 8.19 ( 7.53 - 8.89 ) | 8.01 ( 3526.21 ) | 7.99 ( 7.45 ) | 3 ( 2.88 ) |
| Stent placement* | 572 | 199.29 ( 182.8 - 217.28 ) | 194.48 ( 101315.57 ) | 179.02 ( 166.53 ) | 7.48 ( 7.36 ) |
| Angina pectoris* | 537 | 51.07 ( 46.84 - 55.68 ) | 49.93 ( 25198.16 ) | 48.86 ( 45.45 ) | 5.61 ( 5.48 ) |
| Dizziness* | 452 | 2.36 ( 2.15 - 2.59 ) | 2.34 ( 347.97 ) | 2.33 ( 2.16 ) | 1.22 ( 1.09 ) |
| Cerebrovascular accident* | 351 | 5.43 ( 4.88 - 6.03 ) | 5.36 ( 1245.67 ) | 5.35 ( 4.9 ) | 2.42 ( 2.26 ) |
| Dyspnoea* | 334 | 1.52 ( 1.36 - 1.69 ) | 1.51 ( 57.74 ) | 1.51 ( 1.38 ) | 0.59 ( 0.43 ) |
| Off label use | 300 | 0.9 ( 0.81 - 1.01 ) | 0.9 ( 3.13 ) | 0.9 ( 0.82 ) | -0.15 ( -0.31 ) |
| Fall* | 290 | 2.23 ( 1.99 - 2.51 ) | 2.22 ( 194.51 ) | 2.22 ( 2.01 ) | 1.15 ( 0.98 ) |
| Intentional product use issue* | 284 | 8.38 ( 7.46 - 9.43 ) | 8.3 ( 1817.95 ) | 8.27 ( 7.5 ) | 3.05 ( 2.88 ) |
| Cardiac disorder* | 255 | 6.95 ( 6.14 - 7.87 ) | 6.89 ( 1281.69 ) | 6.87 ( 6.2 ) | 2.78 ( 2.6 ) |
| Nausea | 254 | 0.83 ( 0.73 - 0.94 ) | 0.83 ( 8.97 ) | 0.83 ( 0.75 ) | -0.27 ( -0.45 ) |
| Diabetes mellitus* | 244 | 8.06 ( 7.1 - 9.14 ) | 7.99 ( 1487.53 ) | 7.96 ( 7.16 ) | 2.99 ( 2.81 ) |
| Malaise* | 239 | 1.35 ( 1.19 - 1.53 ) | 1.35 ( 21.52 ) | 1.35 ( 1.21 ) | 0.43 ( 0.24 ) |
| Constipation* | 226 | 2.77 ( 2.43 - 3.16 ) | 2.75 ( 252.39 ) | 2.75 ( 2.46 ) | 1.46 ( 1.27 ) |
| Drug ineffective | 224 | 0.42 ( 0.37 - 0.48 ) | 0.43 ( 173.01 ) | 0.43 ( 0.39 ) | -1.22 ( -1.41 ) |
| Headache | 210 | 0.85 ( 0.74 - 0.97 ) | 0.85 ( 5.44 ) | 0.85 ( 0.76 ) | -0.23 ( -0.43 ) |
| Product use issue* | 195 | 2.69 ( 2.34 - 3.1 ) | 2.68 ( 205.67 ) | 2.68 ( 2.38 ) | 1.42 ( 1.21 ) |
| Cardiac operation* | 192 | 61.85 ( 53.56 - 71.43 ) | 61.36 ( 11097.13 ) | 59.75 ( 52.97 ) | 5.9 ( 5.69 ) |
| Hypoacusis* | 187 | 11.21 ( 9.7 - 12.95 ) | 11.13 ( 1716.24 ) | 11.08 ( 9.82 ) | 3.47 ( 3.26 ) |
| Cardiac failure congestive* | 182 | 5.55 ( 4.8 - 6.43 ) | 5.52 ( 672.8 ) | 5.51 ( 4.87 ) | 2.46 ( 2.25 ) |
| Hypertension* | 179 | 2.18 ( 1.88 - 2.53 ) | 2.17 ( 113.85 ) | 2.17 ( 1.92 ) | 1.12 ( 0.9 ) |
| Seizure* | 161 | 3.64 ( 3.12 - 4.26 ) | 3.63 ( 306.24 ) | 3.62 ( 3.18 ) | 1.86 ( 1.63 ) |
| Asthenia | 160 | 1.09 ( 0.94 - 1.28 ) | 1.09 ( 1.27 ) | 1.09 ( 0.96 ) | 0.13 ( -0.1 ) |
| Pneumonia* | 159 | 1.27 ( 1.09 - 1.49 ) | 1.27 ( 9.1 ) | 1.27 ( 1.11 ) | 0.34 ( 0.11 ) |
| Hypotension* | 152 | 1.95 ( 1.67 - 2.29 ) | 1.95 ( 70.35 ) | 1.95 ( 1.7 ) | 0.96 ( 0.73 ) |
| Fatigue | 146 | 0.47 ( 0.4 - 0.56 ) | 0.48 ( 84.43 ) | 0.48 ( 0.42 ) | -1.07 ( -1.3 ) |
| Wrong technique in product usage process* | 135 | 1.69 ( 1.43 - 2 ) | 1.68 ( 37.69 ) | 1.68 ( 1.46 ) | 0.75 ( 0.5 ) |
| Tremor* | 132 | 2.04 ( 1.72 - 2.42 ) | 2.03 ( 69.16 ) | 2.03 ( 1.76 ) | 1.02 ( 0.77 ) |
| Unevaluable event* | 129 | 4.17 ( 3.5 - 4.95 ) | 4.15 ( 308.01 ) | 4.14 ( 3.58 ) | 2.05 ( 1.8 ) |
| Memory impairment* | 125 | 2.28 ( 1.91 - 2.72 ) | 2.27 ( 89.26 ) | 2.27 ( 1.96 ) | 1.18 ( 0.93 ) |
| Atrial fibrillation* | 117 | 3.09 ( 2.57 - 3.7 ) | 3.07 ( 163.86 ) | 3.07 ( 2.64 ) | 1.62 ( 1.35 ) |
| Vomiting | 117 | 0.65 ( 0.54 - 0.78 ) | 0.65 ( 22.3 ) | 0.65 ( 0.56 ) | -0.62 ( -0.89 ) |
| Cardiac pacemaker insertion* | 111 | 47.9 ( 39.67 - 57.83 ) | 47.68 ( 4967 ) | 46.7 ( 39.89 ) | 5.55 ( 5.27 ) |
| Dementia* | 109 | 10.42 ( 8.63 - 12.58 ) | 10.37 ( 919.28 ) | 10.33 ( 8.82 ) | 3.37 ( 3.09 ) |
| Cardiac failure* | 108 | 3.45 ( 2.86 - 4.17 ) | 3.44 ( 187.01 ) | 3.44 ( 2.93 ) | 1.78 ( 1.5 ) |
| Cardiac arrest* | 107 | 3.36 ( 2.78 - 4.07 ) | 3.35 ( 176.71 ) | 3.35 ( 2.86 ) | 1.74 ( 1.47 ) |
| Syncope* | 107 | 2.74 ( 2.27 - 3.32 ) | 2.73 ( 117.73 ) | 2.73 ( 2.33 ) | 1.45 ( 1.17 ) |
| Coronary artery disease* | 105 | 8.67 ( 7.16 - 10.51 ) | 8.64 ( 706.6 ) | 8.61 ( 7.33 ) | 3.11 ( 2.82 ) |
| Pulmonary embolism* | 103 | 2.75 ( 2.26 - 3.34 ) | 2.74 ( 113.91 ) | 2.74 ( 2.33 ) | 1.45 ( 1.17 ) |

Abbreviation: Asterisks (*) indicate statistically significant signals in algorithm; ROR, reporting odds ratio; PRR, proportional reporting ratio; EBGM, empirical Bayesian geometric mean; EBGM05, the lower limit of the 95% CI of EBGM; IC, information component; IC025, the lower limit of the 95% CI of the IC; CI, confidence interval; PT, preferred term.
